# Conformational Dynamics of the Activated GLP-1 Receptor-G_s_ Complex Revealed by Cross-Linking Mass Spectrometry and Integrative Structure Modeling

**DOI:** 10.1021/acscentsci.3c00063

**Published:** 2023-04-24

**Authors:** Shijia Yuan, Lisha Xia, Chenxi Wang, Fan Wu, Bingjie Zhang, Chen Pan, Zhiran Fan, Xiaoguang Lei, Raymond C. Stevens, Andrej Sali, Liping Sun, Wenqing Shui

**Affiliations:** † iHuman Institute, ShanghaiTech University, Shanghai 201210, China; ‡ School of Life Science and Technology, ShanghaiTech University, Shanghai 201210, China; § University of Chinese Academy of Sciences, Beijing 100049, China; ∥ Structure Therapeutics, South San Francisco, California 94080, United States; ⊥ National Facility for Protein Science in Shanghai, Shanghai Advanced Research Institute, Chinese Academy of Science, Shanghai 201210, China; # Biocreater (WuHan) Biotechnology Co., Ltd, Wuhan 430075, China; ■ Beijing National Laboratory for Molecular Sciences, State Key Laboratory of Natural and Biomimetic Drugs, Key Laboratory of Bioorganic Chemistry and Molecular Engineering of Ministry of Education, Department of Chemical Biology, College of Chemistry and Molecular Engineering, Peking-Tsinghua Center for Life Sciences, Peking University, Beijing 100871, China; ○ Quantitative Biosciences Institute, University of California, San Francisco, San Francisco, California 94143, United States; ▲ Department of Bioengineering and Therapeutic Sciences, University of California, San Francisco, San Francisco, California 94143, United States; ▽ Department of Pharmaceutical Chemistry, University of California, San Francisco, San Francisco, California 94143, United States

## Abstract

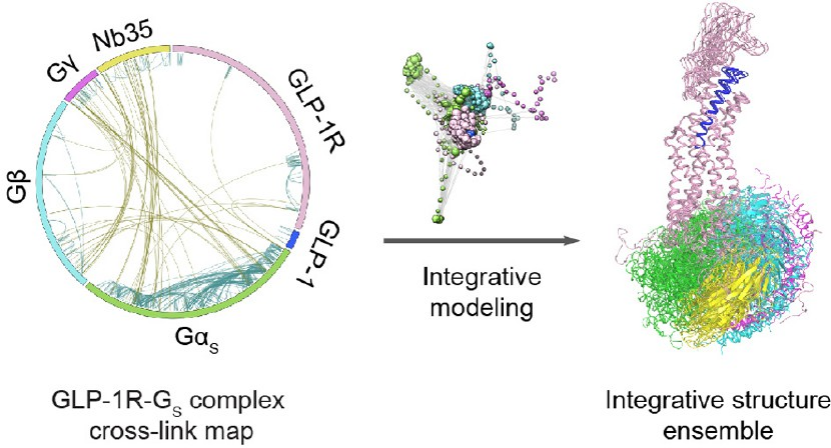

Despite advances in characterizing the structures and functions of G protein-coupled receptors (GPCRs), our understanding of GPCR activation and signaling is still limited by the lack of information on conformational dynamics. It is particularly challenging to study the dynamics of GPCR complexes with their signaling partners because of their transient nature and low stability. Here, by combining cross-linking mass spectrometry (CLMS) with integrative structure modeling, we map the conformational ensemble of an activated GPCR-G protein complex at near-atomic resolution. The integrative structures describe heterogeneous conformations for a high number of potential alternative active states of the GLP-1 receptor–G_s_ complex. These structures show marked differences from the previously determined cryo-EM structure, especially at the receptor–G_s_ interface and in the interior of the G_s_ heterotrimer. Alanine-scanning mutagenesis coupled with pharmacological assays validates the functional significance of 24 interface residue contacts only observed in the integrative structures, yet absent in the cryo-EM structure. Through the integration of spatial connectivity data from CLMS with structure modeling, our study provides a new approach that is generalizable to characterizing the conformational dynamics of GPCR signaling complexes.

## Abstract 2

A combination of cross-linking mass spectrometry with integrative structure modeling allows mapping of the conformational ensemble of an activated GPCR-G protein complex at near-atomic resolution.

## Introduction

G protein-coupled receptors (GPCRs) are integral membrane proteins that share a common seven-transmembrane (TM) topology.^1^ GPCRs mediate the majority of cellular responses to external stimuli, including light, odorants, hormones, and growth factors, which makes them the most frequent drug target family.^2^ An activating ligand, or agonist, stabilizes a GPCR conformation that interacts with a heterotrimeric G protein to promote the exchange of GTP for GDP from the Gα subunit. GTP-bound Gα dissociates from the GPCR and Gβγ subunits, and Gα and Gβγ separately mediate downstream signaling activities.^2,3^ In the simplified GPCR free energy landscape, most receptors bound to an inverse agonist or certain receptors in an unliganded state exist primarily in a low-energy inactive state. Agonist binding induces receptor conformational changes to promote the formation of an intermediate state that is primed for interactions with its cytoplasmic signaling partners, such as heterotrimeric G proteins.^2^ G protein coupling further lowers the energy level of the intermediate state to stabilize the receptor in a fully active conformation, which is observed in many structures determined for agonist-bound GPCR complexes with G proteins.^2,3^

However, GPCRs do not switch abruptly from an inactive state to an intermediate state or an active state upon agonist and G protein binding. Instead, they exist in thermal equilibrium among different states by sampling multiple conformations.^2,4^ An agonist shifts the conformational landscape toward a new equilibrium in which more molecules can bind the partner G protein, while G protein engagement significantly increases the population of active conformations or introduces new conformations.^2,4^ A long-standing challenge to decoding the molecular basis of receptor activation is to structurally characterize multiple coexisting conformational states and uncover new functional states of GPCRs and their signaling partners and to understand how the structural dynamics contribute to pharmacological activities of different ligands and different receptors.^2,4,5^

A growing number of GPCR structures, either in inactive or active conformations, have been determined because of the technical breakthroughs in structural biology. However, these structures resolved by cryo-electron microscopy (cryo-EM) or X-ray free-electron lasers represent static snapshots of the most stable states and cannot reveal conformations of more transient states, such as metastable or transition states. Conventionally, NMR, fluorescence and electron paramagnetic resonance spectroscopies, and molecular dynamics (MD) simulations have been employed to uncover the conformational distribution and dynamics of distinct intermediate states.^6−14^ Such studies of the β_2_-adrenergic receptor and other GPCRs strongly imply that the G protein-bound active or intermediate states are more conformationally heterogeneous than the inactive states.^2,4^ Multiple distinct states associated with the active conformation are observed by the aforementioned spectroscopic approaches,^6−9,12,13^ yet they are structurally intractable by crystallography or cryo-EM. Revealing the atomic structures of these alternative activated or intermediate states would provide new insights into the receptor activation mechanism that may support more informed structure-based drug design.

Cross-linking mass spectrometry (CLMS) has become a powerful technique for probing the topology and dynamics of proteins and their complexes. CLMS yields structural information generally inaccessible to conventional structural biology approaches because it can capture transient rearrangements and interactions of proteins that occur in solution or in the cellular environment.^15−17^ By providing spatial restraints that are orthogonal to low-density EM maps and important for integrative structure modeling,^18,19^ CLMS has manifested its value in elucidating the architectures of large protein assemblies, such as 26S proteasome and nuclear pore complex.^20−22^ However, there is a very limited application of CLMS combined with integrative structure modeling or Rosette docking to the structural characterization of alternative conformational states for dynamic protein complexes.^23,24^ CLMS alone can also provide information about conformational dynamics, yet at a low resolution.^15,25^

GLP-1 receptor (GLP-1R) is a prototypical class B GPCR that is predominantly coupled to the stimulatory G protein G_s_ to raise intracellular cAMP levels. Multiple peptide agonists that activate GLP-1R are approved, or in clinical development, for the treatment of type 2 diabetes and/or obesity.^26^ To promote mechanistic understanding and drug development, a number of high-resolution structures have been determined for the active-state GLP-1R-G_s_ complex bound to a peptide or a small-molecule agonist.^27−33^ Although these structures demonstrate marked differences in the extracellular ligand binding pocket, they share similar backbone conformations at the intracellular face where different agonist-bound receptors are associated with the heterotrimeric G_s_. Moreover, the mode of interaction between activated GLP-1R and G proteins is highly conserved across class B GPCRs and shares many charged and hydrophobic contacts at the interface,^34−38^ thereby defining a major structural feature of the fully active states of class B GPCRs. However, it remains unknown whether less stable conformations coexist with the predominant active state of the GLP-1R-G_s_ complex and, if they do, whether they also mediate G protein-dependent signaling.

**Figure 1:**
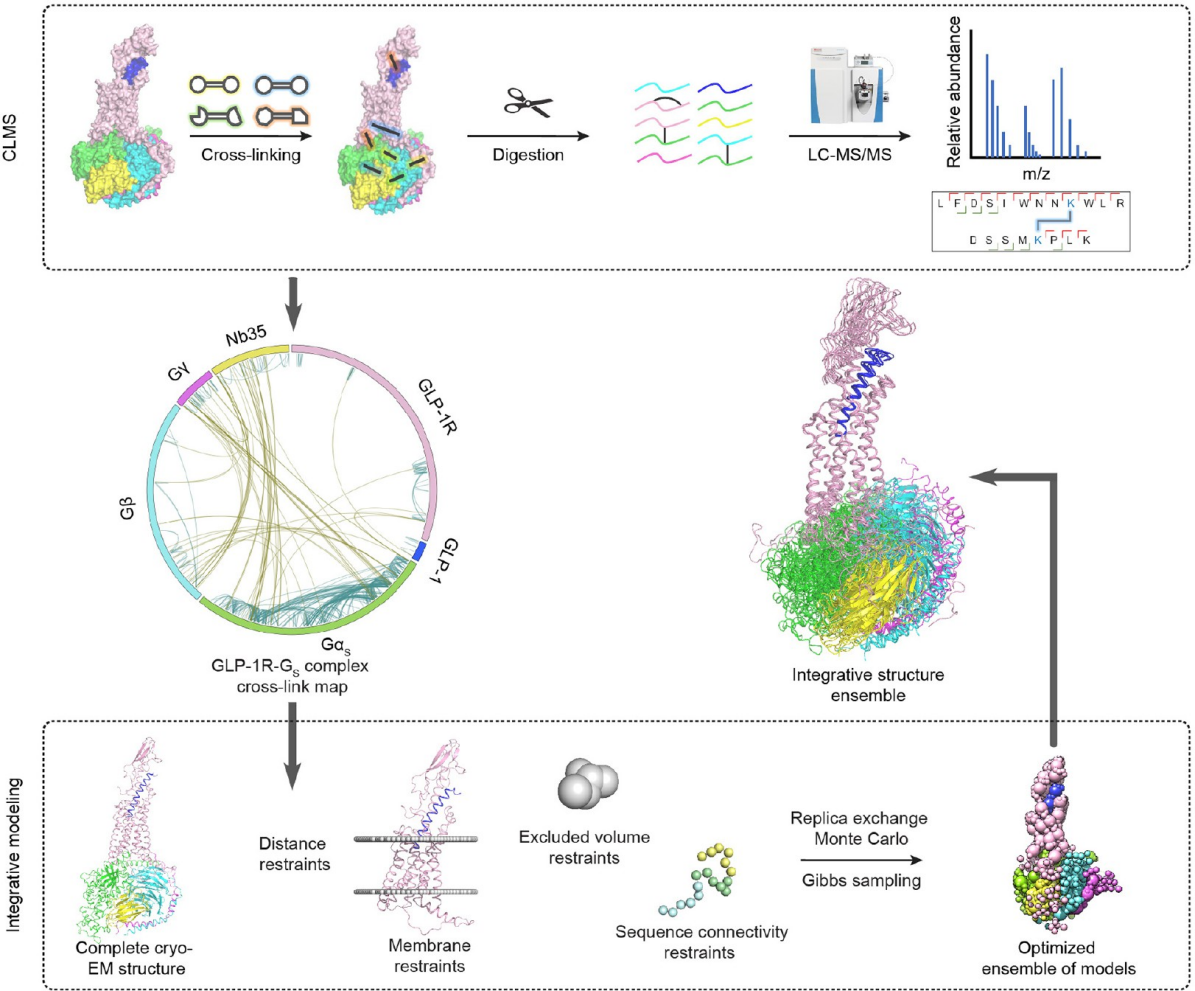
Experimental framework. This framework involves cross-linking mass spectrometry (CLMS) analysis of the GLP-1R-G_s_ protein complex (upper) and integrative modeling (lower). The protein complex was treated by multiple chemical cross-linkers at optimal conditions and subjected to LC-MS/MS analysis, which created a comprehensive cross-link map. This cross-link data set, together with the complete cryo-EM structure, provides input information for integrative structure modeling that computes an ensemble of integrative structures for the GLP-1R-G_s_ complex in solution (also see Supplementary Figure 4 and Supplementary Method).

Herein, we establish an approach to combine CLMS with integrative modeling to map the alternative conformational ensemble of an agonist-bound GLP-1R-G_s_ complex (Figure 1). The resulting integrative structure ensemble suggests a remarkable heterogeneity of the activated GLP-1R-G_s_ complex in solution. The integrative structures describe conformations for a high number of potential alternative active states that are substantially different from the cryo-EM structure, especially at the receptor–G_s_ protein interface and in the interior of the heterotrimeric G_s_ protein. Alanine-scanning mutagenesis coupled with signaling assays validated the functional significance of 24 interface residue contacts exclusively observed in the integrative structures. Thus, our study provides a new approach to probe the conformational dynamics of an active-state GPCR-G protein complex at near-atomic resolution, which would enhance our understanding of GPCR and G protein activation mechanisms.

## Results

### Combinatory Cross-Linking of GLP-1-Bound GLP-1R-G_s_ Complex

As previously described, we achieved comprehensive CLMS analysis of purified GPCR proteins by combined use of several cross-linkers of varied residue reactivity.^39^ Here, we first evaluated five reagents in cross-linking the GLP-1-bound GLP-1R-G_s_ complex. Four selected reagents generated cross-links for amine–amine (BS3, DSG), amine–carboxyl (EDC), and carboxyl–carboxyl (PDH) residue pairs (Supplementary Figure 1a). The recently developed cross-linker KArGO is reactive to the guanidine group and allows for selective cross-linking between Arg and Lys. KArGO has demonstrated robust performance in cross-linking not only soluble proteins but also membrane proteins such as GPCRs.^39,40^

**Figure 2:**
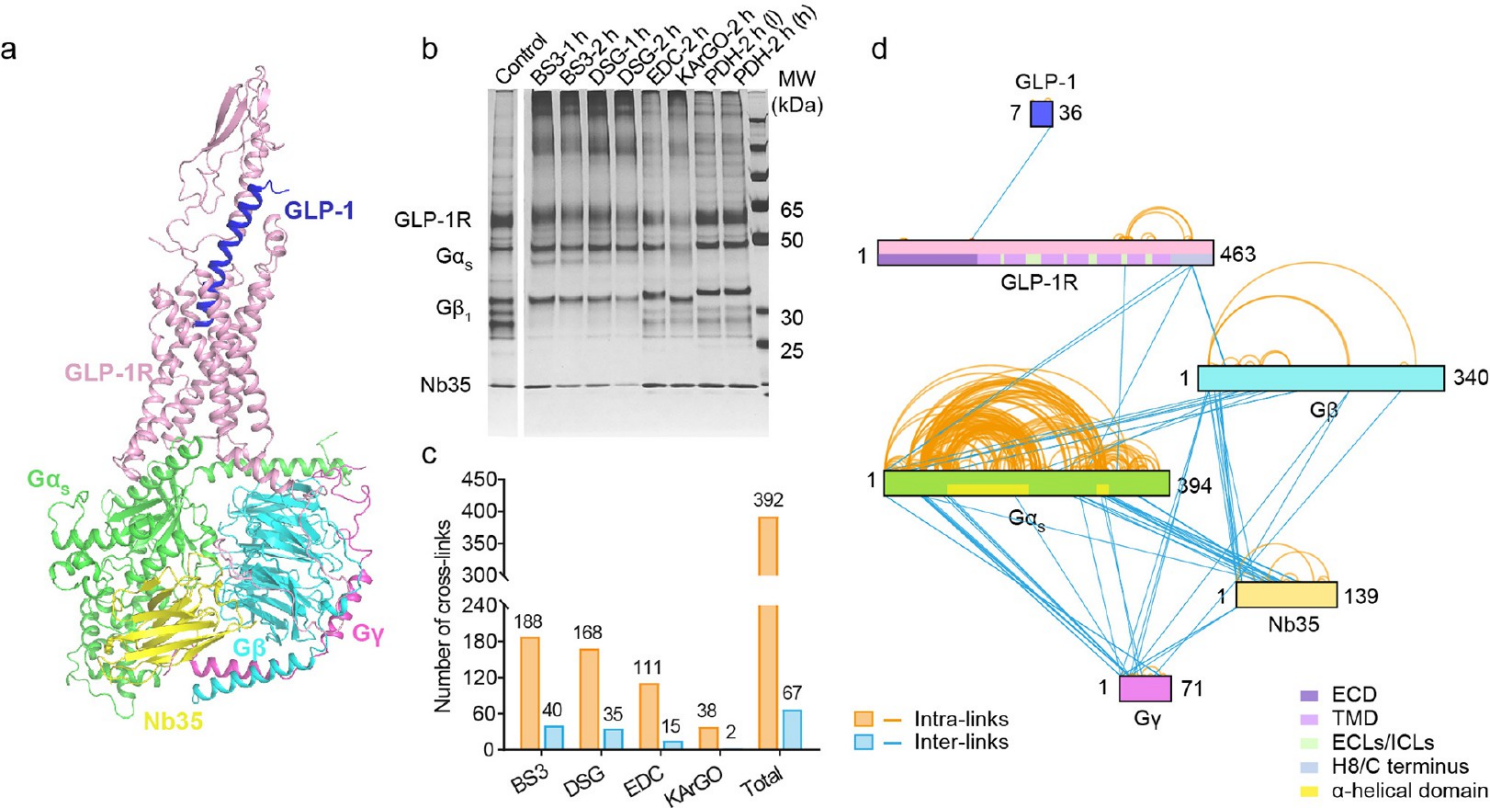
CLMS analysis of the GLP-1R-G_s_ complex. (a) The complete cryo-EM structure of the GLP-1R-G_s_ complex is obtained by modeling segments without density in the cryo-EM map (PDB 6X18). (b) SDS-PAGE image of the GLP-1R-G complex after treatment with five different cross-linkers. Cross-linking conditions, such as different incubation time for each reagent and low (l) or high (h) concentrations for PDH, are annotated. (c) Number of cross-links identified for the protein complex treated by specific cross-linkers. (d) The overall cross-link map for the GLP-1R-G_s_ complex. In (c,d), intra- and intermolecular cross-links are designated in orange and blue, respectively.

Each cross-linker sets an upper bound on the basis of the Euclidean Cα–Cα distance of the cross-linked residues; this threshold corresponds to the sum of the maximal cross-linker length and an additional tolerance of 10 Å that accounts for the lengths of cross-linked side chains and the uncertainty of modeling the backbone positions^41^ (Supplementary Figure 1b). On the basis of these distance thresholds, we calculated the maximal possible numbers of intra- and intermolecular cross-links that can be captured by a given cross-linker type in the reported GLP-1R-G_s_ complex structure. Because the original cryo-EM structure (PDB 6X18)^31^ has a few segments with missing cryo-EM density, we first generated 100 complete structural models (Supplementary Table 1). A centroid model was then derived from these 100 models, referred to as the complete cryo-EM structure of the GLP-1R-G_s_ complex (Figure 2a). On the basis of the theoretical calculation, five cross-linkers yielded distinct and complementary sets of cross-links within the complete cryo-EM structure of the GLP-1R-G_s_ complex (Supplementary Figure 1c,d).

Next, we coexpressed human GLP-1R with Gα_s_, His-Gβ_1_, and Gγ_2_ in *Spodoptera frugiperda* (Sf9) insect cells and induced the formation of an active-state complex by the addition of 10 μM GLP-1 in the presence of apyrase and the nanobody Nb35.^28^ The GLP-1R- G_s_ complex was purified according to the same procedure used for the structural determination of the sample complex.^31^ Purified complexes were resolved as monodispersed peaks on size-exclusion chromatography (SEC) and contained all the expected components (Supplementary Figure 2a). Then, we collected the monodisperse peaks and performed chemical cross-linking of the protein complex with each of the five cross-linkers separately. Both SDS-PAGE and immunoblotting analysis revealed the formation of fully cross-linked products by all cross-linkers at multiple conditions, though PDH generated cross-linking patterns in the high mass range were different from those for the other four cross-linkers (Figure 2b, Supplementary Figure 2b).

Purified GPCRs and their complexes are notoriously unstable when released from the cell membrane, and changes in the chemical environment may result in receptor destabilization, unfolding, and aggregation.^42,43^ Given that cross-linking at certain conditions can perturb protein structures,^44^ we assessed the impact of different cross-linking treatments on the monodispersity of the GLP-1R-G_s_ complex. The majority of cross-linked complexes maintained their monodispersity, although a marginal fraction of aggregates was observed at some conditions (Supplementary Figure 2c).

### Identification and Structural Mapping of Chemical Cross-Links

All cross-linked GLP-1R-G_s_ complexes were subjected to proteolysis and LC-MS/MS analysis for cross-link peptide identification (Figure 1 upper). CLMS analysis of each cross-linking product yielded a distinct set of reproducible cross-linked peptides identified in at least two experimental replicates except for the PDH cross-linking samples (Supplementary Table 2). After converging cross-linked peptides into nonredundant residue-to-residue cross-links, we identified a total of 392 intra- and 67 intermolecular cross-links derived from different types of cross-linking products (Figure 2c). Between BS3 and DSG products, 47.1% of linkages overlap, whereas the remaining 52.9% of linkages are unique to either cross-linker (Supplementary Figure 3a). Therefore, combinatory CLMS analysis with multiple cross-linkers enabled cross-validation and created the most extensive cross-link map of the active-state GLP-1R-G_s_ complex.

In this cross-link map, most intramolecular cross-links are found within the Gα_s_, Gβ_1_, or Gγ_2_ subunit, whereas about 10% of cross-links are present within the GLP-1R protein (Figure 2d, Supplementary Figure 3b). The intermolecular cross-links are mainly observed between different G protein subunits, while many fewer cross-links were identified between GLP-1R and Gα_s_/Gβ_1_ subunits (Figure 2d, Supplementary Figure 3b). The lower number of cross-links detected in GLP-1R than in G protein subunits reflects the challenge of cross-linking membrane proteins, which likely results from fewer cross-linkable residues at the interaction interfaces and weaker detectability of cross-linked peptides^23,39^

**Figure 3:**
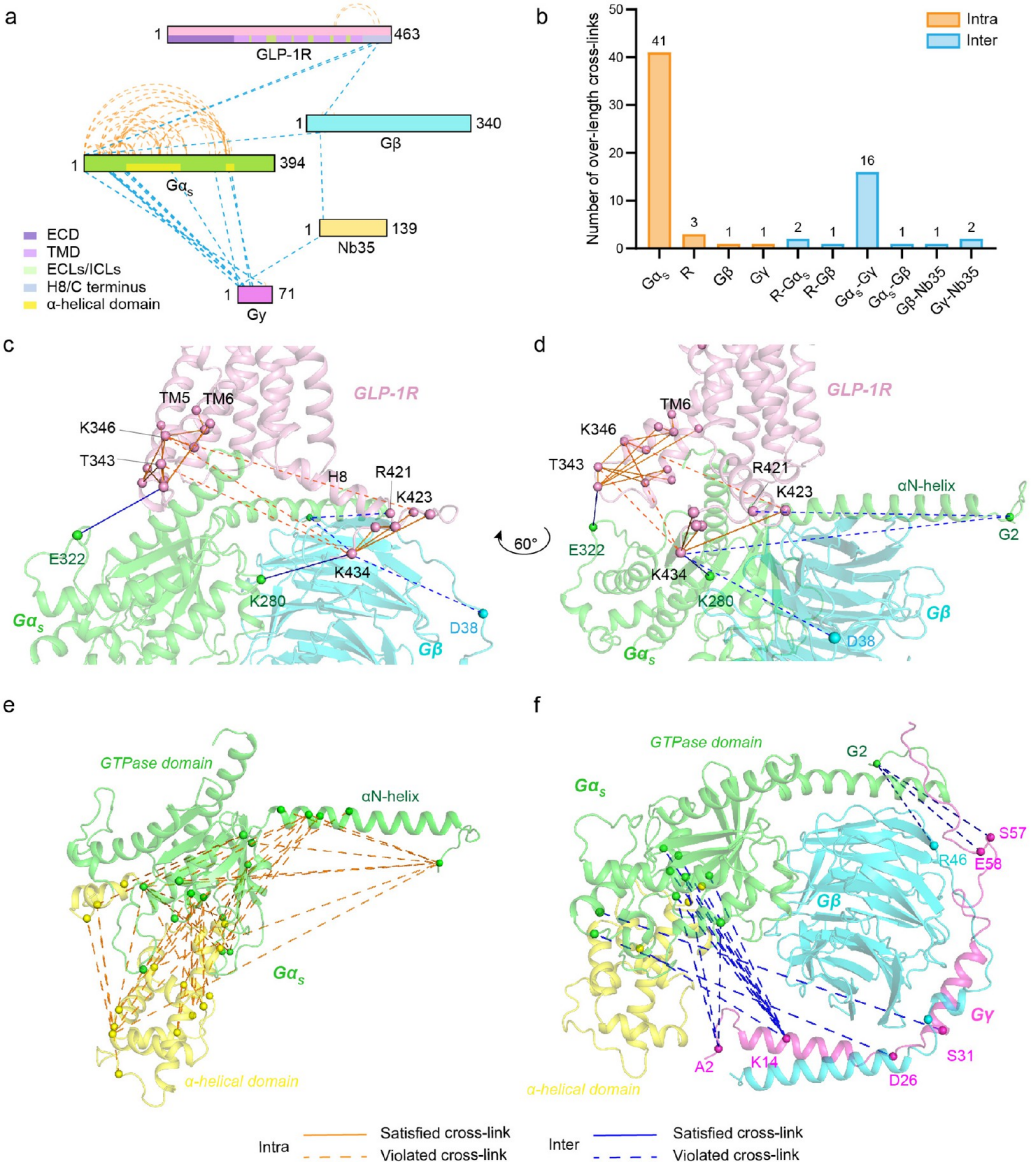
Distribution of cross-links that violate the complete cryo-EM structure of the GLP-1R-G_s_ complex. (a) Map of violated cross-links between or within different subunits. (b) Number of violated cross-links identified in specific subunits. (c–f) Structural mapping of satisfied and violated cross-links within GLP-1R or between GLP-1R and G_s_ subunits (c,d), and violated cross-links within Gα_s_ (e) or between different G_s_ subunits (f). All cross-links are mapped to the complete cryo-EM structure. Intra- and intermolecular cross-links are designated by orange and blue lines (a,c–f) or bars (b).

To assess the consistency of our experimentally identified cross-links with the existing GLP-1R-G_s_ complex structure, we mapped all cross-links to the complete cryo-EM structure (Figure 2a). Because each cross-linker has a specific distance threshold, any cross-link between a residue–residue pair whose Cα–Cα distance in the complete cryo-EM structure exceeds this threshold is considered violated by the spatial connectivity embedded in the complete cryo-EM structure. Among the 69 violated cross-links identified in the GLP-1R-G_s_ complex, 46 are intramolecular cross-links mainly found within G_s_ subunits or GLP-1R. For the violated intermolecular cross-links, the majority are located between different G protein subunits, while three are present at the receptor–G_s_ interface (Figure 3a,b). Representative violated intra- and intermolecular cross-links are illustrated in Figure 3c–f. These violated cross-links may reveal conformational dynamics of the GLP-1R-G_s_ complex in solution and provide essential information for integrative modeling to map the conformational landscape of alternative active states.

### Integrative Structure Modeling of the GLP-1R-G_s_ Complex

To capture alternative conformations of the GLP-1R-G_s_ complex, we performed integrative structure modeling in four stages (Figure 1 lower, Supplementary Figure 4, Supplementary Method, and Supplementary Table 3): (1) the gathering of information, (2) representation of structural components of the GLP-1R-G_s_ complex and translation of information into a scoring function, (3) configurational sampling to produce an ensemble of structures that satisfy the restraints, and (4) analysis and validation of the ensemble structures. Input information included the cross-link data set (Supplementary Table 2) and the complete cryo-EM structure of the GLP-1R-G_s_ complex. The model representation was obtained as follows. First, α-helices and β-sheets were constrained into one rigid body when their conformations were (1) resolved in the complete cryo-EM structure and (2) within the cross-linker distance thresholds. Second, the remaining structures of different components were represented by six rigid bodies corresponding to different domains of GLP1-R and G_s_ subunits. Third, short segments linking rigid bodies and density missing regions, including loops and terminal residues, in the original cryo-EM structure were modeled as flexible strings of beads, with each bead representing an individual residue. The scoring function was a sum of terms that corresponded to cross-links, protein–membrane interactions, sequence connectivity, and excluded volumes. Next, configurations of all seven rigid bodies were exhaustively sampled using a Monte Carlo method starting with a random initial configuration, followed by assessment of the sampling exhaustiveness and structure modeling precision (Supplementary Figure 5).

In total, 844 020 structure models were obtained that sufficiently satisfied the applied restraints. A single distinct cluster containing the majority (89%) of the individual models was identified.^45^ Model precision was quantified by the average Cα RMSD (root mean square deviation) of a model in the cluster to the cluster centroid of 16.9 Å. As expected, the integrative structures in the cluster satisfied the five different types of a cross-link significantly better than the complete cryo-EM structure (85.7–99.4% versus 75.9–90.8%) (Supplementary Figure 6 and Supplementary Table 4). This result suggests that the model ensemble is a better representation of the GLP-1R-G_s_ complex conformations in solution than the cryo-EM structure on its own. Finally, for subsequent analysis, atomic structures were computed for a random subset of 30 000 coarse-grained integrative structures. The centroid structure in the ensemble was defined as the structure that minimizes the sum of the RMSD values against the other structures in the ensemble.

### Conformational Dynamics of the GLP-1R-G_s_ Complex Integrative Structures

**Figure 4:**
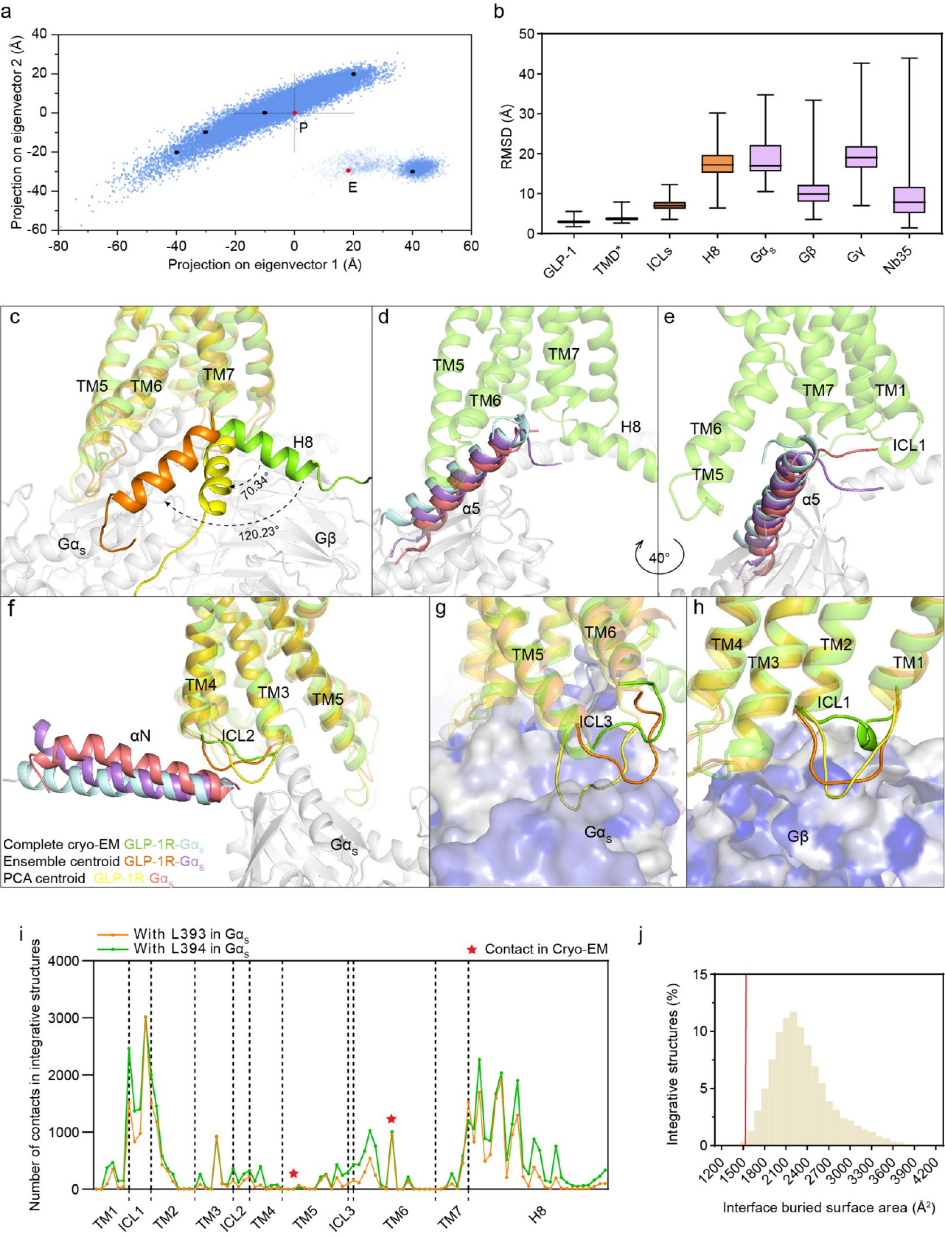
Conformational dynamics of the GLP-1R-G_s_ complex revealed by integrative structures. (a) Principal component analysis (PCA) of 30 000 integrative structures over the mass centers of rigid bodies from the G_s_ heterotrimer after aligning seven transmembrane domains of GLP-1R. Two integrative structure centroids (P, PCA centroid; E, ensemble centroid) are highlighted by red dots, and the other five representative integrative structures are highlighted by black dots. (b) RMSD analysis of specific GLP-1R domains and G protein subunits in 30 000 integrative structures against the complete cryo-EM structure. TMD* includes the seven transmembrane domains, the extracellular domain, and the extracellular loops of GLP-1R. (c–h) Comparison of receptor H8 orientation (c), Gα_s_ α5 helix and N-terminal tail conformations (d,e), Gα_s_ αN helix and receptor ICL2 conformations (f), ICL3 and ICL1 conformations (g,h) at the receptor–G_s_ interface among the complete cryo-EM structure and two integrative structure centroids. Three structures are color-coded, as in (f). The rotation angles of H8 in integrative structures relative to H8 in the cryo-EM structure are annotated in (c). (i) L393 (orange line) and L394 (green line) at the unraveled tail of the Gα_s_ α5 helix make abundant contacts with various residues in different regions of GLP1-R in the integrative structures. In contrast, L393 only makes contacts with two residues in receptor TM5 or TM6 (labeled as red stars) in the complete cryo-EM structure. (j) Distribution of the buried surface areas at the receptor–G_s_ interface in 30 000 integrative structures. The interface buried surface area in the complete cryo-EM structure is indicated by a red line.

To map the conformational landscape of 30 000 integrative structures, we conducted a principal component analysis (PCA) over the mass centers of rigid bodies from the G_s_ heterotrimer after aligning seven transmembrane domains of GLP-1R (Figure 4a). Three clusters were identified from the PCA profile of G_s_ protein, and the PCA centroid structure was defined as the structure located at the center (Figure 4a). At this point, we obtained two centroid structures for subsequent structural comparison: the previously computed ensemble centroid structure and the PCA centroid structure. We randomly selected five additional structures on the basis of the PCA profile, which resulted in a total of seven structures to represent the integrative structure ensemble. These seven representative structures were uploaded into the PDB-Dev, a nascent worldwide Protein Data Bank (wwPDB) archive for integrative structures and associated data^46^ (PDB ID code: PDBDEV_00000200) (Supplementary Figure 7). Additionally, we computed the Cα RMSD of individual subunits of the 30 000 integrative structures against the complete cryo-EM structure to assess their similarity (Figure 4b). While the extracellular and transmembrane domains of GLP-1R in the integrative structures are similar to those in the cryo-EM structure, large variations are found for helix H8 and intracellular loops (ICLs) of GLP-1R and different G_s_ subunits.

To further illustrate the major structural differences between the integrative structures and the complete cryo-EM structure, we aligned the two centroid structures (ensemble centroid and PCA centroid) with the complete cryo-EM structure to specifically compare the receptor H8 and G protein coupling sites. In the complete cryo-EM structure, H8 forms hydrophilic interactions with the Gβ subunit, which is a key difference between class B and class A GPCRs in their G protein-bound active state structures.^28,47,48^ Strikingly, a massive rearrangement of helix H8 is observed in two integrative structures, in which H8 rotates toward the cytoplasmic ends of TM5 and TM6 with an angle of 120° and 70°, respectively, relative to the H8 in the complete cryo-EM structure (Figure 4c). This H8 reorientation spans a wide range from 36° to 168° for all 30 000 integrative structures (Supplementary Figure 8a). The substantial reorientation of H8 results mainly from the satisfaction of several cross-links between H8, the C terminus, and TM6 that are violated by the complete cryo-EM structure (Figure 3c).

In all activated GLP1-R-G_s_ complex structures, the most extensive G protein contacts are formed by the C-terminal α5 helix of Gα_s_, which inserts into the receptor intracellular cavity to make hydrophilic interactions with residues in TM5, TM6, and H8, as well as hydrophobic contacts with TM3.^28,31^ Interestingly, the N-terminal end of the α5 helix of Ga_s_ shifts downward in the integrative structures relative to the complete cryo-EM structure, while its C-terminal end remains anchored to the receptor core. Moreover, the C-terminal end of the α5 helix undergoes partial unwinding in the integrative structures (Figure 4d,e). The unraveled tail of the α5 helix swings so widely that its last two residues (L393 and L394) make new contacts with multiple regions of GLP1-R, especially ICL1 and H8, in contrast to the original contacts between L393 and TM5 or TM6 in the complete cryo-EM structure (Figure 4i). Similarly to the α5 helix, there is a slight reorientation of the N-terminal end of Ga_s_ αN helix in the integrative structures, although its C-terminal end remains in close contact with ICL2 of the receptor (Figure 4f). The downward movement of ICL2 in the integrative structures leads to more extensive interactions with Ga_s_ αN helix. Furthermore, the other two intracellular loops, ICL3 and ICL1, also adopt conformations largely different from those in the complete cryo-EM structure, which could result in more contacts with Gα_s_ and Gβ subunits, respectively (Figure 4g,h). Analysis of the receptor–G_s_ interface areas restricted to ICL1, ICL2, or ICL3 reveals expanded interactions with all three loop regions in 42.4–99.9% of integrative structures compared with the complete cryo-EM structure (Supplementary Figure 8b-d).

Taken together, the integrative structures likely demonstrate marked conformational dynamics, particularly at the receptor–G_s_ heterotrimer interface. As a result, the total buried surface area engaging G protein coupling sites is substantially increased from 1595 Å^2^ in the complete cryo-EM structure to an average of 2367 Å^2^ for integrative structures (Figure 4j).

### Functional Contacts Exclusively Observed in the Integrative Structures

**Figure 5:**
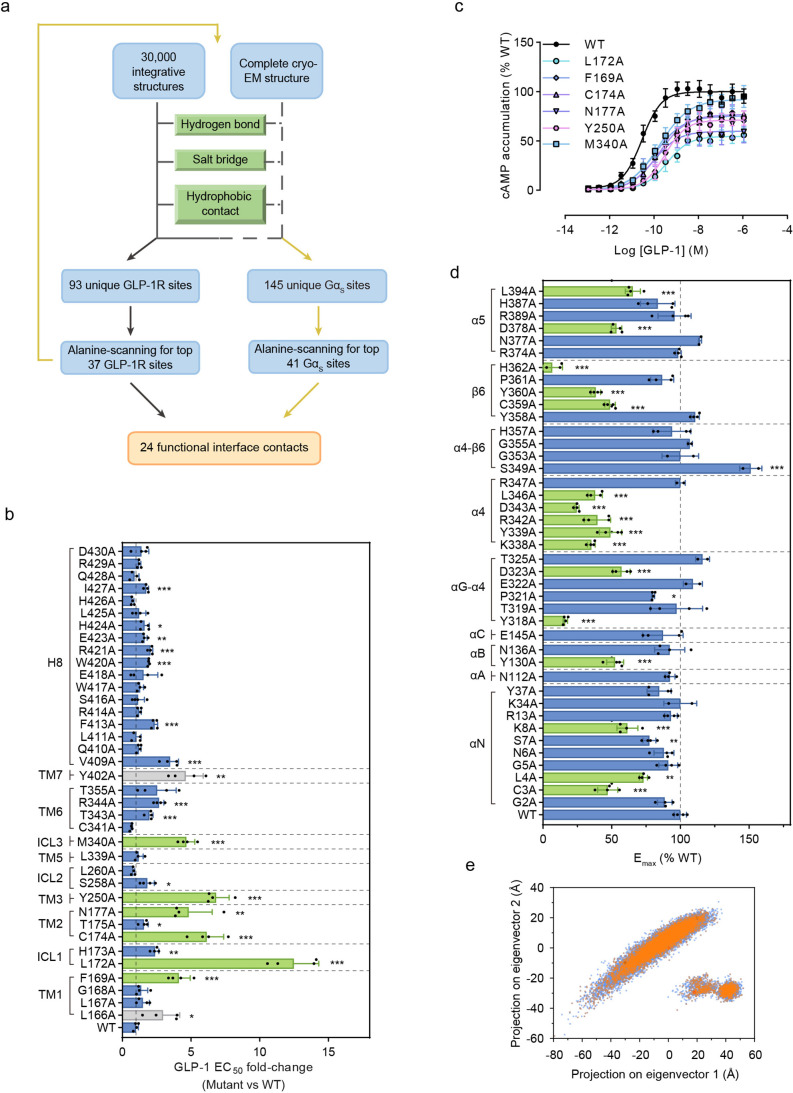
Identification of functional contacts present at the GLP-1R-G_s_ interface of integrative structures yet absent in the cryo-EM structure. (a) Schematic representation of the structural interrogation and experimental validation workflow to identify a total of 24 functional interface contacts exclusively observed in the integrative structures. (b) Alanine-scanning mutagenesis of top 37 unique GLP-1R residues identified 18 mutants with significant reduction of the GLP-1 potency in GLP-1R-mediated cAMP accumulation. The fold-change of GLP-1 EC_50_ determined for cells expressing a mutant vs wild-type (WT) is shown for each mutant. Six mutants with the most profound changes in GLP-1 potency are indicated in green columns. Two mutants with abnormal expression and deficient GLP-1 efficacy are indicated in gray columns. (c) Concentration–response curves of cAMP accumulation for the six mutants highlighted in green in (b). (d) Alanine-scanning mutagenesis of top 41 unique Gα_s_ residues identified 16 mutants (indicated in green) with significant reduction of the GLP-1 efficacy in GLP-1R-mediated G protein activation. GLP-1 *E*_max_ determined for a mutant relative to WT is shown for each mutant. In (b–d), *p < 0.05, **p < 0.01, and ***p < 0.001. See Supplementary Table 6 for EC_50_ and *E*_max_ values. Data represent mean ± SEM of four independent experiments. (e) Distribution of 9683 integrative structures that contain at least 1 of the 24 functional interface contacts in the structure ensemble PCA profile.

The comparison of two centroid structures with the complete cryo-EM structure unveiled distinct features of G protein coupling and H8 orientation in the integrative structures. We then devised a data analysis workflow to systematically validate potential interactions at the receptor–G_s_ heterotrimer interface that are exclusively observed in our integrative structures (Figure 5a). From the 30 000 integrative structures, we identified 4877 interface contacts harboring one receptor residue and one G_s_ protein residue that form at least one type of noncovalent interaction (salt bridge, hydrogen bond, or hydrophobic contact) defined by stringent criteria^49−51^ (Methods). These contacts are mediated by a total of 111 receptor residues and 384 G_s_ protein residues. In parallel, we identified 27 interface contacts from the complete cryo-EM structure of the GLP-1R-G_s_ complex, which are mediated by 18 receptor residues. Comparison of the two sets of receptor residues gave rise to 93 unique contact residues in GLP-1R only observed in the integrative structures and not in the cryo-EM structure (Figure 5a, Supplementary Figure 9a). After ranking the frequency of noncovalent interactions engaged by each unique GLP-1R contact residue, we selected 37 residues with the highest interaction frequency to examine their individual contribution to GLP-1R-mediated cAMP signaling (Supplementary Table 5).

Mutagenesis of each selected contact residue to alanine resulted in a significant reduction of GLP-1 potency in cAMP signaling induction for 18 mutants in comparison with the wild-type (WT) (Figure 5b, Supplementary Table 6). These functional contact residues are located in various structural regions of the receptor, including TM1, TM2, TM3, TM6, H8, ICL1, ICL2, and ICL3. Among them, the substitution of six residues, F169^1.64^, L172^ICL1^, C174^2.44^, N177^2.47^, Y250^3.53^, and M340^ICL3^ (numbers in superscript refer to the Wootten numbering system for class B GPCRs), had the most profound effects on GLP-1 potency in the cAMP accumulation assay (right-shift of EC_50_ by 4.1- to 10.5-fold) and on GLP-1 efficacy in the G protein dissociation assay (Figure 5c, Supplementary Table 6).

To search for Gα_s_ protein residues forming at least one type of the aforementioned noncovalent interactions with these six receptor residues, we reinterrogated 30 000 integrative structures to find 145 unique Gα_s_ contact residues only present in the integrative structures (Figure 5a, Supplementary Figure 9a). Then, 41 Gα_s_ residues with the highest interaction frequency were selected to examine their individual contributions to GLP-1R-mediated G_s_ protein activation (Supplementary Table 7). The substitution of each selected contact residue to alanine resulted in a significant reduction of GLP-1 efficacy in G_s_ activation for 16 mutants in comparison with WT (Figure 5d). These mutants displayed above 89% total G_s_ protein expression relative to WT (Supplementary Figure 9b). The contact residues with functional impact are located in multiple Gα_s_ domains, such as the N-terminal loop, the α4 helix, and the β6 β-sheet, that do not make direct interactions with the receptor in the cryo-EM structure (Figure 5d, Supplementary Figure 10).

Altogether, we identified 24 functional contact pairs harboring 22 distinct residues at the receptor–Gα_s_ protein interface for which a mutation of either residue in a contact pair impacts the GLP-1R-mediated cAMP signaling or G protein activation (Supplementary Table 8). None of these 22 functional residues are engaged in any aforementioned interactions (salt bridge, hydrogen bond, or hydrophobic contact) with neighboring residues (Supplementary Figure 10). A total of 9683 integrative structures containing at least one of the 24 functional interface contacts are widely distributed in the structure ensemble PCA profile, thereby implying the high structural diversity of these alternative active states of the GLP-1R-G_s_ complex (Figure 5e).

**Figure 6:**
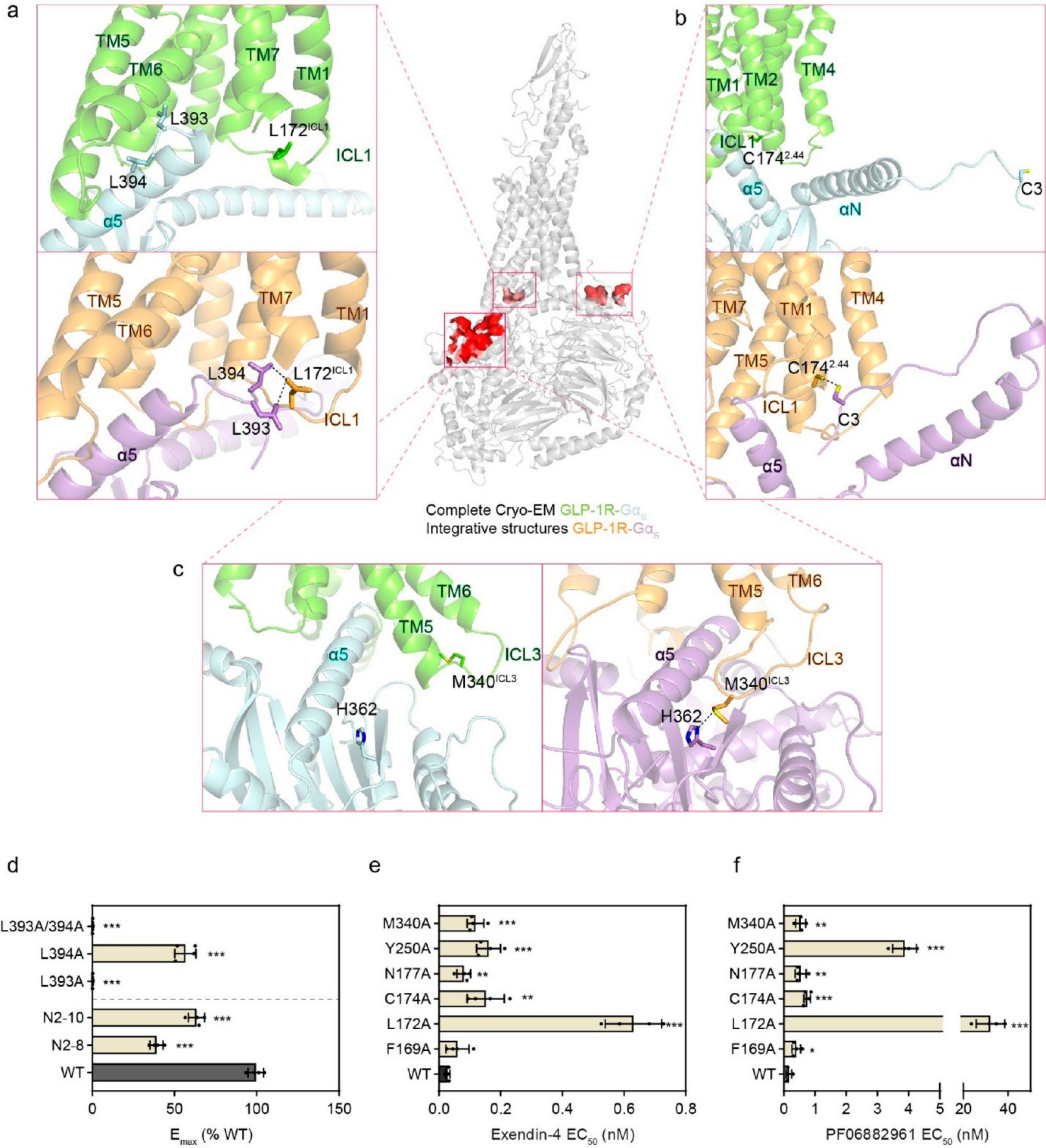
Comparison of functional residue conformations in the complete cryo-EM structure vs representative integrative structures. (a–c) Illustration of three residue pairs making no contacts in the complete cryo-EM structure [upper in (a,b), left in (c)] yet forming functional contacts in representative integrative structures [lower in (a,b), right in (c)]. The functional residue pairs (one from GLP1-R, the other from Gα_s_) selected for comparison are L172^ICL1^ and L393/L394 (a), C174^2.44^ and C3 (b), and M340^ICL3^ and H362 (c). They are shown as sticks with residue interactions annotated as dashed lines in integrative structures. All identified functional contacts are located in three distinct regions at the receptor–Gα_s_ interface, as shown by the functional Gα_s_ residues labeled in red in the complete cryo-EM structure (center). (d) The GLP-1 efficacy in GLP-1R-mediated G protein activation determined for single or double mutants (upper), and truncation mutants of the N-terminal 2–8 and 2–10 residues (lower) for Gα_s_ relative to WT. (e,f) The potency of exendin-4 (e) and PF06882961 (f) in GLP-1R-mediated cAMP accumulation determined for receptor mutants and WT. In (d–f), *p < 0.05, **p < 0.01, and ***p < 0.001. See Supplementary Figure 12 for EC_50_ and *E*_max_ values. Data represent mean ± SEM of four independent experiments.

More specifically, these 24 functional contacts harboring six receptor residues are mapped to three distinct regions at the receptor–Gα_s_ interface. First, in 3761 integrative structures, F169^1.64^ and L172^ICL1^ close to or within ICL1, or Y250^3.53^ at the cytoplasmic end of TM3, could form hydrophobic contacts with L393 or L394 in the C-terminal tail of the α5 helix (Figure 6a, Supplementary Table 8). By contrast, L394 does not mediate interactions with any residues in the complete cryo-EM structure. Individual replacement of L394 to the smaller alanine side chain caused a decrease in GLP-1 efficacy in inducing G protein dissociation, and substitution of both L394 and L393 completely abolished the GLP-1 activity (Figure 6d, Supplementary Figure 9c,d). This result indicates the importance of the two leucine residues at these positions to mediate dynamic contacts with the receptor that seems necessary for G protein activation. Second, two functional residues adjacent to ICL1 (C174^2.44^ and N177^2.47^) could interact with C3 and K8 in the N-terminal loop of the Gα_s_ subunit through a hydrogen bond in 68 integrative structures (Figure 6b, Supplementary Table 8). Notably, the Gα_s_ N-terminal loop lacks density in many cryo-EM maps of activated GLP-1R-G_s_ complexes,^28,31,52,53^ which indicates its high flexibility. Our integrative modeling captures the possible conformations of this loop linked to a slightly bent αN helix through the satisfaction of the initially violated cross-links between the Gα_s_ N terminus and H8 or C terminus of GLP-1R or other regions in Gα_s_ (Figure 3d,e). The functional importance of the Gα_s_ N-terminal loop is further corroborated by the truncation of the first 2–8 and 2–10 residues that both significantly attenuated the G_s_ protein activation mediated by GLP-1R (Figure 6d, Supplementary Figure 9d,e). Third, M340 in the ICL3 of GLP1-R could form unique hydrogen bonds with 13 functional residues in different Gα_s_ domains in 7126 integrative structures, which implies the prevalent dynamics of ICL3 and its enhanced interactions with the Gα_s_ subunit in the alternative active states of the GLP-1R-G_s_ complex (Figure 6c, Supplementary Table 8).

As the six functional residues of GLP-1R discovered in this study are highly conserved across species (Supplementary Figure 11), we tested whether they also mediate intracellular signaling induced by other agonists. Mutagenesis of these residues, except for F169^1.64^, led to attenuated cAMP signaling by both another peptide agonist (exendin-4)^52^ and a small-molecule agonist (PF 06882961)^31^ compared with WT, while substitution of F169^1.64^ significantly impaired the potency of the small-molecule agonist (Figure 6e,f, Supplementary Figure 12). These results suggest the functional contacts and conformational dynamics observed in the integrative structures may also apply to different agonist-bound GLP-1R-G_s_ complexes.

## Discussion

Despite recent advances in GPCR structural biology, our understanding of GPCR signaling and modulation would not be complete without the complementary information on GPCR conformational dynamics.^4^ Compared with single receptors, the transient nature and low stability of GPCR complexes with their signaling partners have made them particularly more challenging to study dynamics.^4^ Here, by combining CLMS analysis with integrative structure modeling, we establish a new approach to describe the conformational heterogeneity of alternative active states of the GLP-1R-G_s_ complex in solution, which are markedly different from the structure of the same complex determined by cryo-EM.

While integrative structure modeling is commonly employed to depict the molecular architecture of large and heterogeneous systems, such as the ∼52 MDa Nuclear Pore Complex,^18^ our study uniquely reveals the structural dynamics of a relatively small protein complex (165 kDa) at near atomic precision. The high precision of our integrative structures is made possible by the use of four chemical cross-linkers with complementary residue reactivity. Although we obtained the most comprehensive cross-link map of the GLP-1R-G_s_ complex, the number of identified cross-linked peptides and the coverage of the receptor cross-linked sequences still lag behind those for soluble proteins. Future development of new chemical cross-linkers and optimization of the cross-linking procedure are expected to improve the CLMS data set size and quality and, thus, increase the accuracy and precision of integrative modeling of membrane protein complexes. It is also noteworthy that the cross-link map does not reveal direct contacts in the protein complex: instead, they reflect spatial connectivity between different residues.

A hallmark of GPCR activation is the outward movement of TM6 that is accompanied in class B GPCRs by the formation of a sharp kink in the middle of the helix.^47,48^ This prominent structural feature, as well as the overall backbone conformation of the transmembrane bundle observed in the cryo-EM structures of activated GLP-1R-G_s_ complexes, are largely conserved in integrative structures generated in this study. Moreover, the integrative structures satisfy the cross-links significantly better than the cryo-EM structure (85.7–99.4% for integrative structures versus 75.9–90.8%). Thus, the integrative structure ensemble likely represents alternative active-state conformations that may be less stable and exist in subpopulations compared with the dominant active conformation revealed by cryo-EM. These alternative active states mainly differ from the cryo-EM structure at the receptor–G_s_ interface and in the interior of the heterotrimeric G_s_ protein (Figure 4b). Specifically, Gα_s_ exhibits substantial conformational dynamics, as suggested by cross-links violated by the cryo-EM structure and satisfied by at least some of the integrative structures: 41 internal cross-links and 19 cross-links between Gα_s_ and receptor or other G_s_ subunits, which jointly account for 87% of all cross-links violated by the cryo-EM structure (Figure 3b). It is known that the Gα subunit undergoes a large structural rearrangement upon receptor binding by breaking and reorganizing a network of contacts within Gα or at the receptor–Gα interface.^54^ Our integrative structures may indicate alternative conformations of the metastable Gα_s_ states that are being activated during interactions with GLP-1R.

In regard to the GLP1-R-G_s_ interface, we observed partial unwinding and swinging of the C terminal end of the Gα_s_ α5 helix, tilting of the N termini of Gα_s_ α5 helix and αN helix, downward movement of three ICLs, and a large reorientation of H8 relative to the cryo-EM structure (Figure 4c–h). It is well recognized that the C terminus of the Gα_s_ α5 helix undergoes a disorder-to-order transition during G protein activation by receptor binding.^54^ Thus, our integrative structures capture Gα_s_ conformations with the C-terminal tail of the α5 helix partially unraveled and making new contacts with multiple receptor domains, which possibly represents certain transition states, before the α5 helix completely folds and inserts into the receptor cytoplasmic cavity.

Some of these conformational dynamics seen in our integrative structures are also reported in structures determined for GLP1-R and other GPCR-G protein complexes. Divergence at the receptor ICLs and the N-terminal region of the Gα_s_ α5 helix is observed in the cryo-EM structures of GLP-1R-G_s_ complexes bound to different peptide agonists.^52^ Moreover, titling of the N termini of Gα_s_ α5 and αN helices is found in the structure of a noncanonical state of the NTSR1-G_i1_ complex.^55^ In the APJR-G_i_ complex structure and active-like AT2R structures, both the H8 of one APJR protomer and H8 of AT2R adopt noncanonical conformations by inverting their orientations and placing their C termini close to the cytoplasmic ends of TM5 and TM6,^56,57^ similarly to the ensemble centroid of our integrative structures (Figure 4c). Therefore, the structural characteristics of alternative active states or transition states observed in our study may be common to multiple activated GPCR signaling complexes.

Our study has several limitations. First, it is technically challenging to experimentally verify the large number of integrative structures generated by this approach. Although we validated some unique functional contacts by alanine-scanning mutagenesis, those contacts without functional validation may still occur in a minor population of less stable conformations. Second, while the integrative structures may represent alternative active or active-like states of the GLP-1R-G_s_ complex in solution, they do not describe a temporal trajectory of structural changes during GLP-1R activation. Third, because of the lack of violated cross-links detected within the ligand binding pocket, our integrative structures do not reveal the structural dynamics of the GLP-1 peptide or its binding site. In the future, an increase in the number of cross-links for all regions in the structure may shed light on the orthosteric pocket dynamics that could be coupled to the receptor–G protein interface dynamics through allosteric communication.^52,54^

In summary, the substantial conformational dynamics of an activated GPCR-G protein complex revealed in this study provides insights into the GPCR activation pathway, as well as opportunities for the design of ligands targeting alternative active states of potential therapeutic values. Furthermore, our approach can also be applied to study the dynamics of other GPCR complexes with different ligands or different partners, thereby, in turn, paving a way for addressing fundamental questions in GPCR biology, such as partial and biased agonism.

## Methods

### Constructs and Insect Cell Expression

The human full-length GLP-1R was cloned into the pFastbac 1 vector with its native signal peptide replaced by hemeagglutinin (HA) to enhance receptor expression, and followed by a FLAG-tag at the N terminus. A tobacco etch virus protease (TEV) site and an 8xHis tag were appended to the C terminus of GLP-1R. A dominant-negative Gα_s_ (DNGαs)^28^ was also cloned into the pFastbac 1 vector. Human Gβ_1_ and human Gγ_2_ were cloned into the pFastBac Dual vector. GLP-1R, DNGα_s_, and Gβ_1_Gγ_2_ were coexpressed at the ratio of 2:2:1 in *Spodoptera frugiperda* (Sf9) insect cells (Invitrogen) at a density of 2 × 10^6^ cells per mL using the Bac-to-Bac Baculovirus system (Invitrogen). Cell pellets were collected by centrifugation after 48 h transfection and stored at −80 °C.

### GLP-1R-G_s_ Complex Purification

The 1 L cell pellets were lysed in 20 mM HEPES (pH 7.4), 50 mM NaCl, and 2 mM MgCl_2_ supplemented with EDTA-free protease inhibitor cocktail tablets. The GLP-1-bound GLP-1R-G_s_ complex was formed in the cell membranes by the addition of 10 μM GLP-1 (GenScript), 10 μg/mL of Nb35-His, 25 mU (milli-units)/mL of apysase (NEB), and 100 μM tris(2-carboxyethyl)phosphine (TCEP) (Thermo Scientific) with incubation for 2 h at 4 °C. The sample was centrifugated at 35 000 rpm for 30 min, and the precipitate was solubilized by 20 mM HEPES (pH 7.4), 100 mM NaCl, 2 mM MgCl_2_, 10 μM GLP-1, 25 mU/mL of apysase, 20 μM TCEP, and 0.5% (w/v) lauryl maltose neopentyl glycol (LMNG, Anatrace) supplemented with 0.1% (w/v) cholesteryl hemisuccinate (CHS, Anatrace) for 2 h at 4 °C. The sample was centrifuged at 35 000 rpm for 30 min, and the supernatant was incubated with 1 mL of TALON resin (Clontech), 20 mM imidazole, 10 μM TCEP, and 1 μM GLP-1 overnight at 4 °C. The resin was washed with 30 column volumes of buffer 1 (20 mM HEPES pH 7.4, 100 mM NaCl, 30 mM imidazole, 0.02% LMNG, 0.004% CHS, 2 mM MgCl_2_, 5 μM GLP-1, and 25 μM TCEP) and 15 column volumes of buffer 2 (20 mM HEPES pH 7.4, 100 mM NaCl, 50 mM imidazole, 0.02% LMNG, 0.004% CHS, 2 mM MgCl_2_, 5 μM GLP-1, and 25 μM TCEP) and followed by elution of buffer 3 (20 mM HEPES pH 7.4, 100 mM NaCl, 300 mM imidazole, 0.01% LMNG, 0.002% CHS, 2 mM MgCl_2_, 30 μM GLP-1, and 25 μM TCEP). The complex was then concentrated by an Amicon Ultra Centrifugal Filter (MWCO, 100 kDa) and separated from contaminants by size-exclusion chromatography on a Superdex 200 Increase 10/300 column (GE Healthcare) with buffer 4 [20 mM HEPES pH 7.4, 100 mM NaCl, 2 mM MgCl_2_, 0.00075% (w/v) LMNG, 0.00025% (w/v) CHS, and 100 μM TCEP]. The fractions containing the intact GLP-1R-G_s_ complex were collected and pooled.

### Chemical Cross-Linking and Proteolysis

BS^3^, DSG, EDC, and Sulfo-NHS were purchased from Thermo Scientific. PDH and DMTMM were purchased from Sigma. KArGO was provided by Dr. Xiaoguang Lei’s lab. The purified GLP-1R-G_s_ complex was concentrated to 1 mg/mL and incubated with specific chemical cross-linkers at different conditions for 1 or 2 h at 4 °C. For BS^3^, DSG, and KArGO, the cross-linking conditions were 0.5 mM BS^3^, 0.5 mM DSG, and 0.1 mM KArGO, which were all prepared in a buffer of 20 mM HEPES pH 7.4, 100 mM NaCl, 2 mM MgCl_2_, 0.00075% (w/v) LMNG, and 0.00025% (w/v) CHS. For EDC, the cross-linking condition was 2 mM EDC and 3 mM Sulfo-NHS mixed in the buffer, with the pH adjusted to 6.5. For PDH, the cross-linking condition was 12.5 mM (low) or 25 mM (high) PDH with DMTMM at the same concentration in the aforementioned buffer (pH 7.4). All reactions were quenched by desalting with a PD Spintrap G-25 column (GE Healthcare) and lyophilized by a SpeedVac concentrator (Labconco).

In the CLMS experiment, five to seven independent cross-linking replicates were prepared for each cross-linker, and all reactions proceeded for 2 h. Each cross-linking product was subjected to proteolysis and LC-MS/MS analysis. The protein samples were first redissolved in 8 M urea (Sigma) and diluted to 1.5 M urea with 20 mM ammonium bicarbonate (Sigma). Then, the protein samples were reduced by 5 mM TCEP for 20 min at 25 °C and alkylated by 10 mM iodoacetamide (Sigma) for 20 min at 25 °C in the dark. Further, the samples were digested with trypsin (Promega) at a ratio of 50:1 protein/trypsin for 12–14 h at 37 °C and were desalted by UltraMicro Spin Column, Silica C18 (The Nest Group). Finally, the peptide samples were lyophilized by SpeedVac concentrator and redissolved by 0.1% formic acid (FA) before LC-MS/MS analysis.

### LC-MS/MS Analysis

Peptide samples from cross-linking products were analyzed using an EASY-nLC 1200 system (Thermo Fisher Scientific) coupled to a Q Exactive HF mass spectrometer (Thermo Fisher Scientific). Samples were loaded onto an analytical column (200 mm × 75 μm) in-house-packed with C18-AQ 1.9 μm C18 resin (Dr. Maisch, Gmbh, Germany) at a flow rate of 300 nL/min with mobile phase A of 0.1% (v/v) FA and mobile phase B of 80% (v/v) acetonitrile (ACN)/0.1% (v/v) FA. Peptides were separated with a 120 min LC gradient (5–45% mobile phase B in 110 min, 45–100% mobile phase B in 5 min, and 100% mobile phase B in 5 min). Full MS scans were acquired at a resolution of 120 000 with an automatic gain control (AGC) target of 3 × 10^6^ and a mass range of 400–1800 *m*/*z*. The Top-N 20 ions with 3+ to 8+ charge states were selected for HCD MS2 fragmentation at a resolution of 30 000 with an AGC target of 1 × 10^5^.

The MS/MS raw data were processed by pLink 2.3.9 for cross-linked peptide identification.^58^ Search parameters for different chemical cross-linkers are defined as below: BS^3^, lysine, serine, threonine, tyrosine or protein N terminus for both α and β sites, 138.068 Da for linker mass, and 156.079 Da for mono mass; DSG, lysine, serine, threonine, tyrosine or protein N terminus for both α and β sites, 96.021 Da for linker mass, and 114.032 Da for mono mass; EDC, lysine or protein N terminus for α site and glutamic acid or aspartic acid for β site, −18.011 Da for linker mass, and 0.000 Da for mono mass; KArGO, lysine or protein N terminus for α site and arginine for β site, 334.084 Da for linker mass, and 352.094 Da for mono mass; and PDH, glutamic acid or aspartic acid for both α and β sites, 152.106 Da for linker mass, and 170.117 Da for mono mass. Other common parameters included the following: enzyme, trypsin; up to 3 missed cleavages; peptide mass, 600–6000 Da; peptide length, 6–60; precursor tolerance, 10 ppm; fragment tolerance, 20 ppm; fixed modification, carbamidomethylation of cysteine; and variable modification, oxidation of methionine. Cross-link peptides identified with <5% FDR and and *E* value < 0.001 in at least two experimental replicates were retained for further analysis.

### Generation of the Complete cryo-EM Structure

The complete cryo-EM structure of the GLP-1-R-Gs complex was built using MODELER 10.2^1^ (Supplementary Figure 2a). First, unstructured components of up to 40 residues were identified as density-missing regions in the cryo-EM structure (PDB code 6X18) (Supplementary Table 2). Second, 100 independent runs were performed to complete the unstructured components. Each run generated 20 models, followed by molecular dynamics refinements. Third, 100 structural models were selected as the ones with the lowest Discrete Optimized Protein Energy (DOPE) score in individual runs. At last, the complete cryo-EM structure was determined as the centroid of these 100 structures with the lowest RMSDs for heavy atoms against all other structures.

### Integrative Modeling

Integrative structure modeling was carried out to determine the alternative active-state structures of the human GLP-1R-Gs complex. Details are provided in the Supporting Information and Supplementary Figure 4.

### Mutagenesis

The cDNA of human GLP-1R was cloned into vector pcDNA 3.1 or pRK. In the cAMP assay, the GLP-1R sequence comprises the native signal peptide (residues 1–23) and a FLAG tag. In flow cytometry analysis, the GLP-1R sequence contains the same native signal peptide, followed by a FLAG or HA tag. Mutations or deletions for the cAMP assay and flow cytometry were generated using seamless cloning with KOD-Plus-Neo (TOYOBO) and confirmed by DNA sequencing.

### cAMP Accumulation Assay and Flow Cytometry

HEK-293T cells were plated in 6-well dishes at a density of 500 000–600 000 cells per well, cultured in DMEM supplemented with 10% (v/v) fetal bovine serum, and maintained in the incubator at 37 °C and 5% CO_2_. After overnight of culture, specified amounts of wild-type or mutant GLP-1R and 0.5 μg of pGloSensor cAMP plasmid were cotransfected into HEK-293T cells using calcium phosphate. After 24 h of culture, the transfected cells were seeded into 384-well plates (12 000 cells per well). cAMP accumulation was measured using the GloSensor cAMP assay kit (Promega) according to the manufacturer’s instructions. In brief, transfected cells were incubated for 30 min in GloSensor cAMP reagent at 37 °C and room temperature. Wild-type or mutant GLP-1R were stimulated with different concentrations of GLP-1 for 15 min at 25 °C and were measured by an EnVision multimode plate reader (PerkinElmer).

To evaluate the cell surface expression of wild-type and mutant GLP-1R, the remaining cells after seeding into 384-well plates were incubated with anti-FLAG M2-FITC antibody (Sigma) or anti-HA–FITC antibody (Sigma) for 30 min at 4 °C and were measured by Beckman Coulter CytoFLEX flow cytometer.

### TRUPATH Biosensor

We used the BRET2-based TRUPATH biosensor system^59^ to monitor G protein activation upon an agonist treatment. Specifically, HEK-293T cells were plated in 6-well dishes at a density of 500 000–600 000 cells per well, cultured in DMEM supplemented with 10% (v/v) fetal bovine serum, and maintained in the incubator at 37 °C and 5% CO_2_. Cells were transfected 18 h later at a 1:3:3:3 DNA ratio of receptor/Gα-RLuc8/Gβ/Gγ-GFP2 using calcium phosphate. Cells were harvested and plated in poly-l-lysine-coated white, clear-bottomed 96-well assay plates (Corning) 24 h after transfection at a density of 30 000–50 000 cells per well and cultured in DMEM supplement with 1% (v/v) dialyzed FBS.

One day after plating in 96-well assay plates, white backings were applied to the plate bottom, and the growth medium was replaced immediately with 60 μL of freshly prepared assay buffer [1 × Hank’s balanced salt solution (HBSS), 20 mM HEPES, pH 7.4, 5 μM coelenterazine 400a (Nanolight Technologies)]. Cells were treated with 20 μL of each compound. Plates were then read in an LB940 Mithras plate reader (Berthold Technologies) with 395 nm (RLuc8-coelenterazine 400a) and 510 nm (GFP2) emission filters at an integration of 0.8 s per well. BRET2 ratios were computed as the ratio of the GFP2 emission to RLuc8 emission.

### Identification of Residue Contacts at the GLP-1R-G_s_ interface

We interrogated 30 000 integrative structures in search of residue–residue contacts at the interface between GLP-1R and G_s_ protein. A residue pair (one from GLP-1R, the other from a G_s_ subunit) is defined as a residue–residue contact if and only if the residue pair can potentially form a hydrogen bond, a salt bridge, or a hydrophobic contact according to the following criteria: (1) A hydrogen bond is defined when the distance between a probable donor and acceptor with an electronegative atom (N, O, S) is smaller than or equal to 3.5 Å. (2) A salt bridge is defined when the distance between a positively charged residue (K, R, H) and a negatively charged residue (D, E) is smaller than or equal to 4.0 Å.^49^ (3) A hydrophobic contact is defined when the distance between two hydrophobic residues is within 4.0 Å,^51^ or the distance between the atoms in one residue and the center of the benzene ring in another aromatic residue is smaller than or equal to 4.0 Å. The distance cut-offs used here are generally accepted empirical values according to previous GPCR structural studies.^28,50,54^ The interface contacts are also obtained from the complete cryo-EM structure of the GLP-1R-G_s_ complex using the same criteria. Both main-chain and side-chain interactions were considered when computing residue contacts from structures. The 24 interface functional contacts identified from integrative structures only involve side-chain interactions to be consistent with the mutagenesis data.

### Interface Buried Surface Area Calculation

The interface area was calculated by the program FreeSASA 2.0 using the Sharke–Rupley algorithm with a probe radius of 1.4 Å and in accordance with the definition of buried surface area in PDBePISA (https://www.ebi.ac.uk/pdbe/pisa/pi_tips.html)

### Statistical Analysis

All pharmacological data are presented as mean ± standard error of the mean (SEM). Statistical analysis was performed using GraphPad Prism 8.0.2 (GraphPad Software). Concentration–response curves were evaluated with a four-parameter logistic equation. The significance was determined with either two-tailed Student’s *t* test or one-way ANOVA test. Significant difference is accepted at *p* < 0.05.

## Data Availability

The LC-MS/MS raw data generated in this study have been deposited to the ProteomeXchange Consortium via the iProX partner repository with the data set identifier PXD039315 (in ProteomeXchange) and IPX0003164000 (in iProX). A summary of all identified cross-linked peptides and cross-linked residues is available in Supplementary Table 2. Input data, modeling scripts and output results are available at https://github.com/salilab/GLP1R-Gs. The integrative structures are deposited to PDB-Dev (https://pdb-dev.wwpdb.org/), with the PDB ID code PDBDEV_00000200.

## Supporting Information Available

Structures and residue specificity of cross-linkers used in this study, experimental data for GLP-1R-G_s_ complex purification and cross-link identification, structural satisfaction rate calculation, protein expression and concentration–response curve data, representative integrative structures, residue contact distribution, and the general scheme and detailed supplementary methods about integrative structure modeling (PDF)
Supplementary Table 1: Sequence lengths used in this work and in the cryo-EM structure of the GLP-1 receptor-Gs complex (PDB 6X18) (XLSX)
Supplementary Table 2: Summary of the identified cross-linked peptides of the active GLP-1R-Gs complex (XLSX)
Supplementary Table 3: Summary of integrative structure determination of the GLP-1 receptor–G_s_ complex (XLSX)
Supplementary Table 4: The distance mapping and satisfaction of different types of cross-links on the cryo-EM structure and the integrative structure ensemble of the GLP-1R-Gs complex (XLSX)
Supplementary Table 5: Frequency of non-covalent interactions for top 37 unique GLP-1R sites on the receptor–G protein interface from the integrative structures (XLSX)
Supplementary Table 6: Summary of EC_50_ in the cAMP accumulation assay and surface expression of top 37 unique GLP-1R sites from integrative structures (XLSX)
Supplementary Table 7: Frequency of non-covalent interactions for top 41 unique Gα_s_ sites making contacts with the six functional GLP-1R sites from the integrative structures (XLSX)
Supplementary Table 8: Frequency of non-covalent interactions for the 24 functional contacts at the receptor–Gα_s_ protein interface of integrative structures (XLSX)
