# Supplementary material for: Conformational Dynamics of the Activated GLP-1 Receptor-Gs Complex Revealed by Cross-Linking Mass Spectrometry and Integrative Structure Modeling
Source: ACS Cent Sci. 2023 Apr 24;9(5):992–1007. doi: 10.1021/acscentsci.3c00063 (PMC10214531; doi:10.1021/acscentsci.3c00063)
Supplement: Supplementary file 1 — oc3c00063_si_001.pdf [file oc3c00063_si_001.pdf]

Supplementary Information for

Conformational dynamics of the activated GLP-1 receptor-G_s complex revealed by cross-linking mass spectrometry and integrative structure modeling

Shijia Yuan[1,2,3‡], Lisha Xia[1,2,3‡], Chenxi Wang[1,2,3‡], Fan Wu[4], Bingjie Zhang[1], Chen Pan[5], Zhiran Fan[6], Xiaoguang Lei[7], Raymond C. Stevens[1,2,4], Andrej Sali[8,9,10], Liping Sun[1*], Wenqing Shui[1,2*]

[1]iHuman Institute, ShanghaiTech University, Shanghai 201210, China

[2]School of Life Science and Technology, ShanghaiTech University, Shanghai 201210, China

[3]University of Chinese Academy of Sciences, Beijing 100049, China

[4]Structure Therapeutics, South San Francisco, CA, USA

[5]National Facility for Protein Science in Shanghai, Shanghai Advanced Research Institute, Chinese Academy of Science, Shanghai 201210, China

[6]Biocreater (WuHan) Biotechnology Co., Ltd, Wuhan 430075, China

[7]Beijing National Laboratory for Molecular Sciences, State Key Laboratory of Natural and Biomimetic Drugs, Key Laboratory of Bioorganic Chemistry and Molecular Engineering of Ministry of Education, Department of Chemical Biology, College of Chemistry and Molecular Engineering, Peking-Tsinghua Center for Life Sciences, Peking University, Beijing 100871, China

[8]Quantitative Biosciences Institute, University of California, San Francisco, San Francisco, CA 94158, USA.

[9]Department of Bioengineering and Therapeutic Sciences, University of California, San Francisco, San Francisco, CA 94158, USA.

[10]Department of Pharmaceutical Chemistry, University of California, San Francisco, San Francisco, CA 94158, USA

‡Equal contribution

*To whom correspondence should be addressed:

Wenqing Shui Email: shuiwq@shanghaitech.edu.cn

Liping Sun Email: sunlp@shanghaitech.edu.cn

Table of Content

Supplementary Fig. 1 | Cross-linkers used in this study and theoretical calculation of cross-links.

Supplementary Fig. 2 | Purification and cross-linking of the GLP-1R-G_s complex.

Supplementary Fig. 3 | Cross-links identified in the GLP-1R-G_s complex.

Supplementary Fig. 4 | The general scheme of integrative structure modeling.

Supplementary Fig. 5 | Validation of the integrative structure ensemble.

Supplementary Fig. 6 | Structural satisfaction rates for different types of cross-linkers based on the complete Cryo-EM structure and integrative structures.

Supplementary Fig. 7 | Representative integrative structures.

Supplementary Fig. 8 | Dynamics of the GLP-1R-Gs interface in integrative structures.

Supplementary Fig. 9 | Identification of $G\alpha_s$ residues important for GLP-1R-mediated G protein activation.

Supplementary Fig. 10 | Contacts present in the complete cryo-EM structure and experimentally verified functional contacts present in the integrative structures.

Supplementary Fig. 11 | Conservativeness of the six functional GLP-1R residues across species.

Supplementary Fig. 12 | GLP-1R-mediated cAMP signaling induced by different agonists.

Supplementary Method

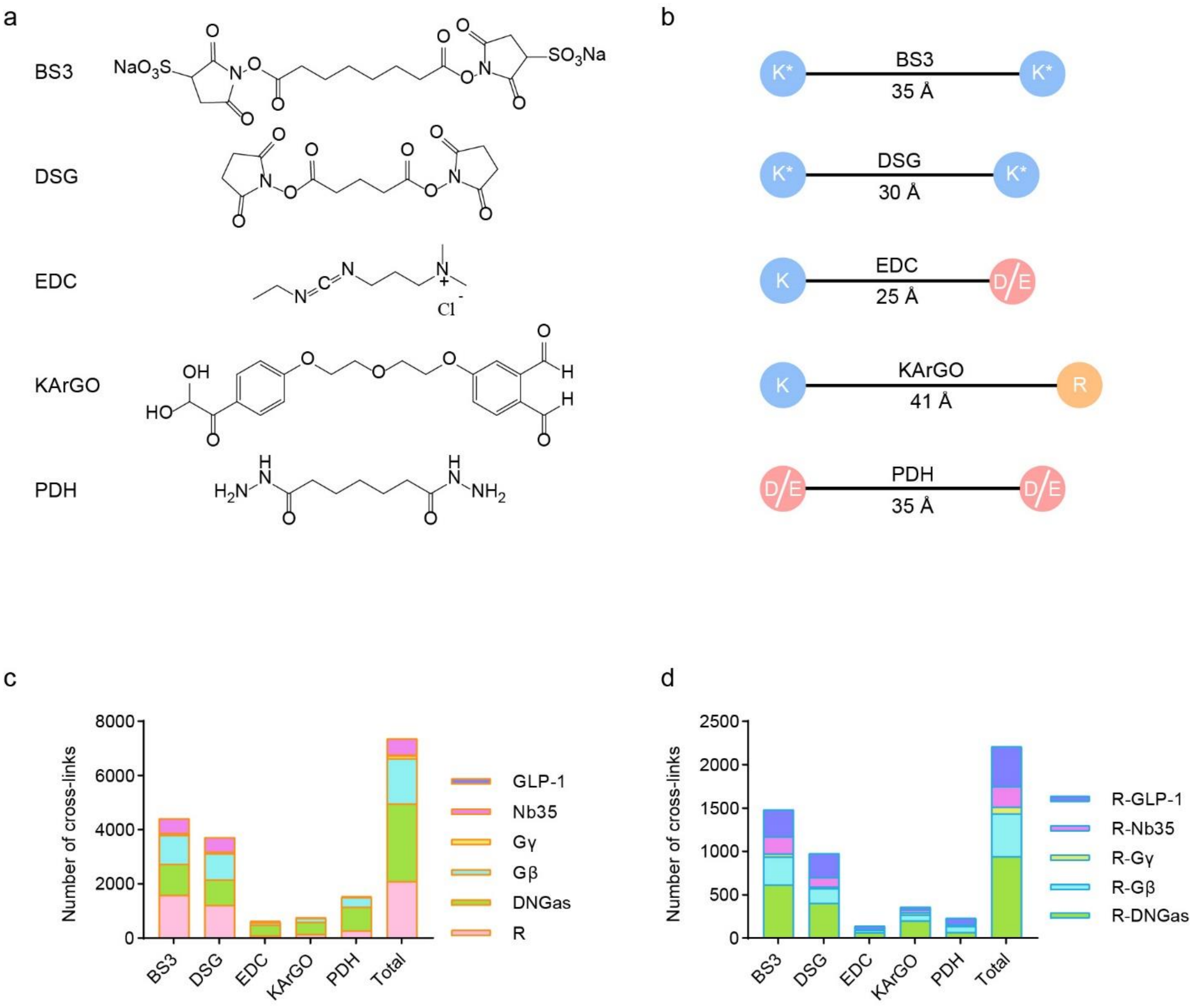

Supplementary Fig. 1 Cross-linkers used in this study and theoretical calculation of cross-links. a Structures of five cross-linkers used in this study. **b** Cross-linking residue specificity and upper bound distance restraint of each cross-linker. K* includes K, Y, S, and T. **c-d** Numbers of theoretically calculated intra (**c**) and inter (**d**) cross-links that can be captured by each cross-linker and all cross-linkers (total) based on the complete cryo-EM structure of the GLP-1R-G_s complex.

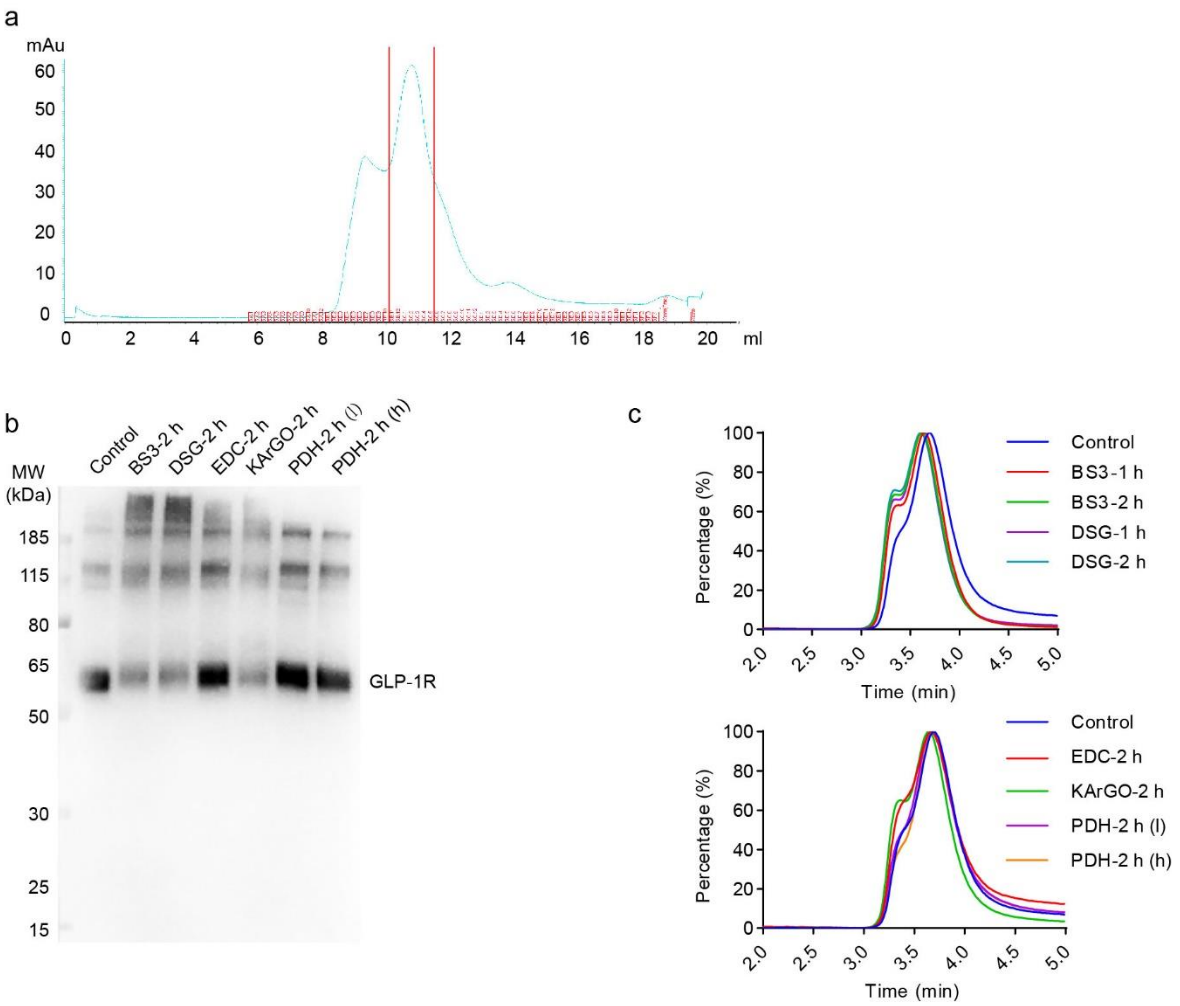

Supplementary Fig. 2 Purification and cross-linking of the GLP-1R-G_s complex. a Size-exclusion chromatography (SEC) profile of the purified complex. Fractions between red lines were collected for the following cross-linking experiment. **b** Immunoblot analysis of the complex after treatment with five different cross-linkers. Proteins were probed with anti-HA antibodies. **c** SEC profiles of the complex after treatment with five different cross-linkers. Cross-linking conditions such as different incubation time for each reagent and low (l) or high (h) concentrations for PDH are annotated.

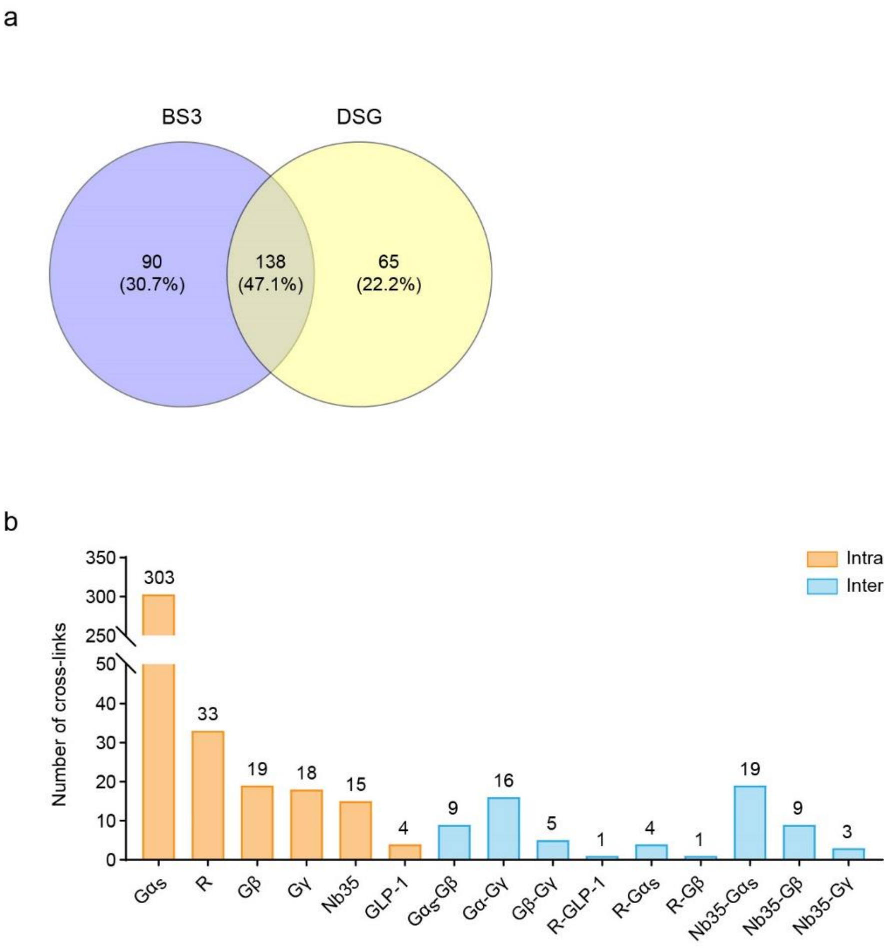

Supplementary Fig. 3 Cross-links identified in the GLP-1R-G_s complex. a Overlap of cross-links identified by BS3 and DSG, which are both amine-amine cross-linkers. **b** Number of intra- and inter-molecular cross-links identified in different subunits of the complex.

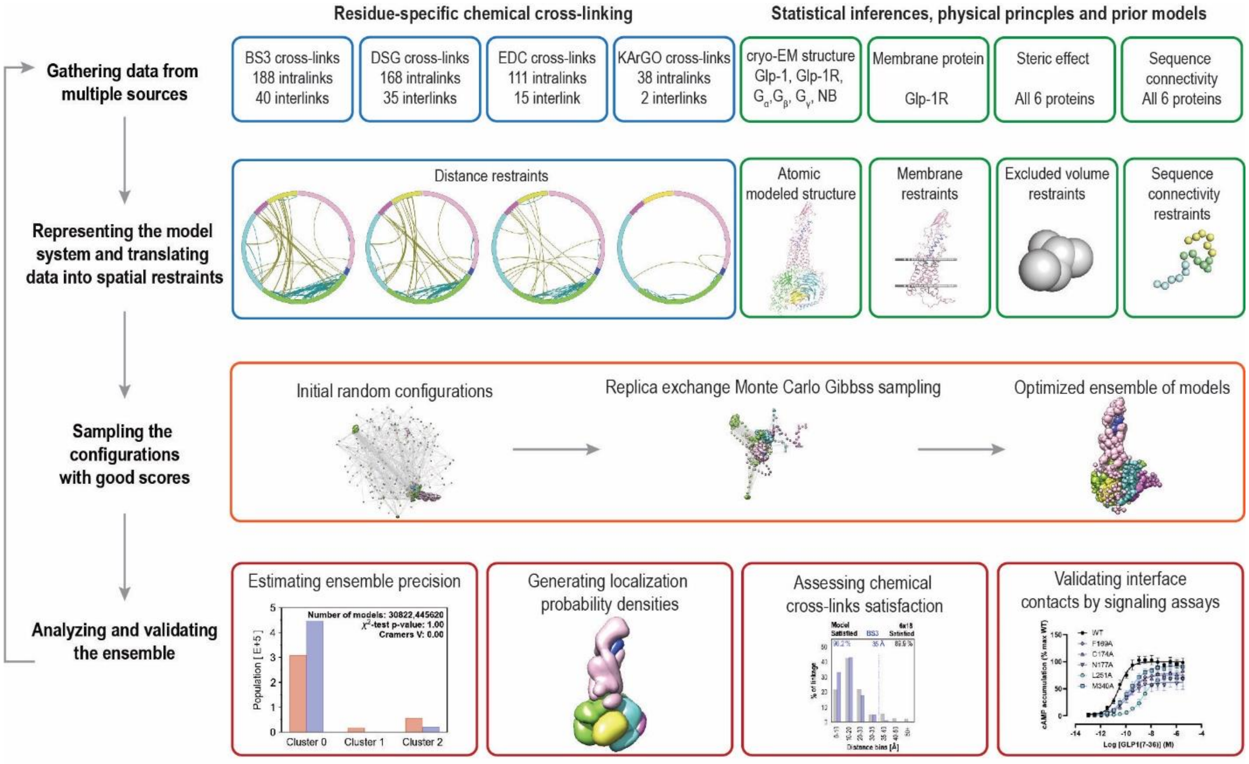

Supplementary Fig. 4 The general scheme of integrative structure modeling. The integrative structure determination of the human GLP-1R-G_s complex proceeds through four stages. First, we gather all information describing the system, including experimental data, statistical inferences, physical principles, and prior models. Second, we represent the system components and translate each piece of input data into a set of spatial restraints. Third, we sample alternative configurations to produce an ensemble of structures with good scores. Fourth, we validate and analyze the ensemble of integrative structures resulting from sampling.

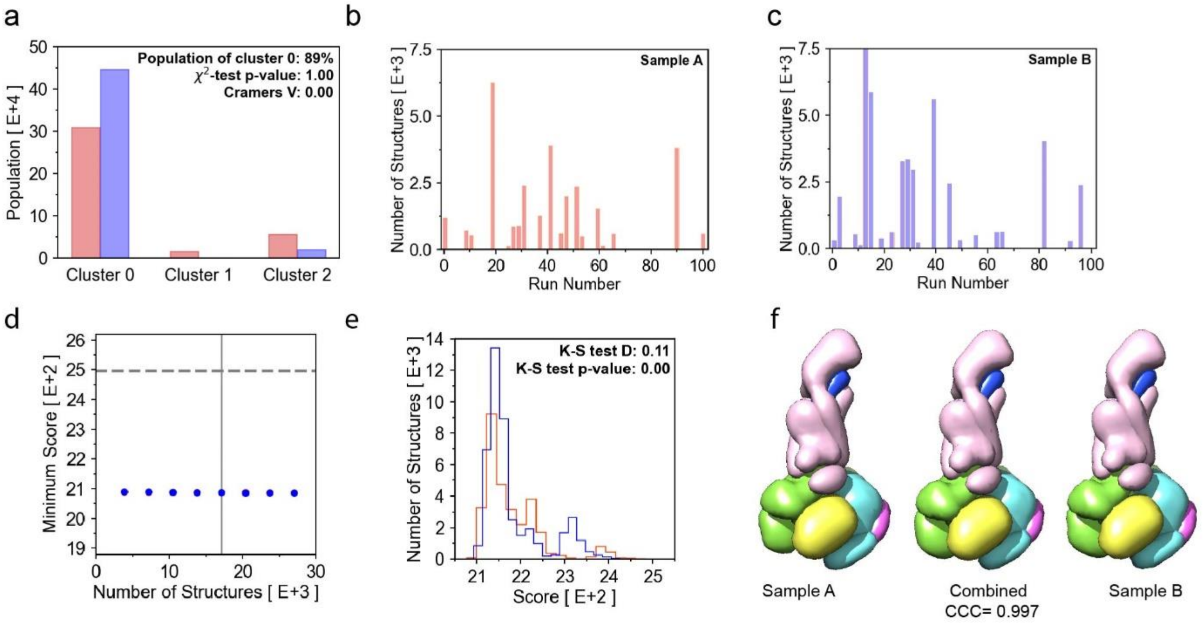

Supplementary Fig. 5 Validation of the integrative structure ensemble. a Number of models in sample A and B in the three clusters obtained by threshold-based clustering using an RMSD threshold of 21 Å. The dominant cluster (cluster 0) contains 89% of the structures. The χ2-test p-value is 1.00 and the Cramer's V is 0.00. **b-c** Number of representative structures randomly selected from individual runs for sample A (red) and B (blue) in cluster 0, respectively. **d** Convergence of the structure score along with the number of selected structures. **e** Distribution of scores for structures in samples A (red) and B (blue). The non-parametric Kolmogorov-Smirnov two-sample test (two-sided) yields a K-S test D of 0.11 and p-value of 0. **f** Comparison of localization probability densities of structures from sample A and B in cluster 0. The cross-correlation coefficient of 0.997 between the localization probability densities of two samples.

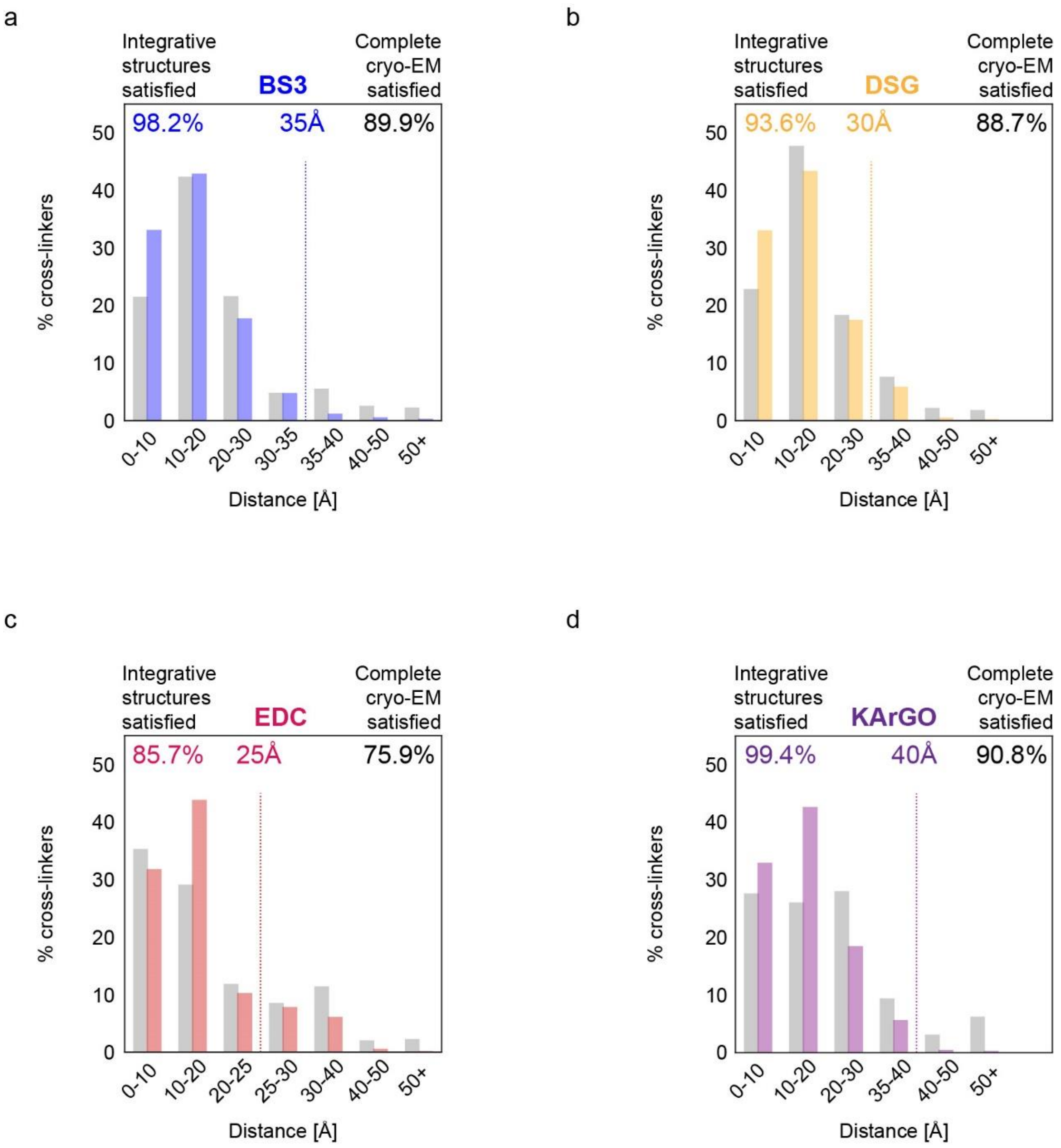

Supplementary Fig. 6 Structural satisfaction rates for different types of cross-linkers based on the complete Cryo-EM structure and integrative structures. Distribution of Cα-Cα distances for cross-links identified with BS3 (**a**), DSG (**b**), EDC (**c**), and KArGO (**d**) based on 30,000 integrative structures (color) and 100 complete structural models of the cryo-EM structure (gray). The distance restraint of each cross-linker is annotated by a dashed line. The rates of cross-links satisfied by the integrative structures or cryo-EM structures are labeled separately.

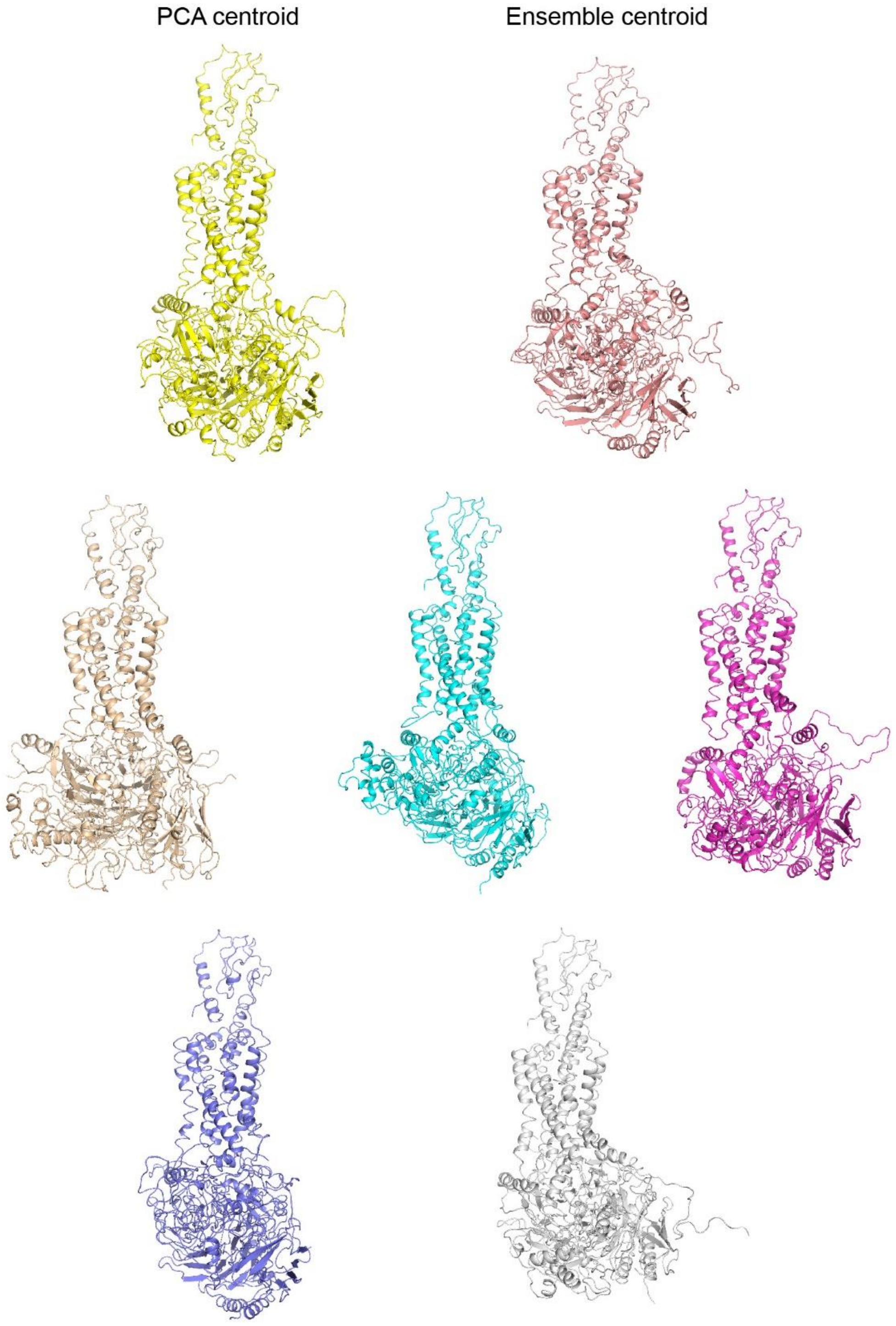

Supplementary Fig. 7 Representative integrative structures. Seven representative structures of the integrative structure ensemble as labeled in the PCA profile (Figure 4a), including the ensemble centroid and PCA centroid, and five other randomly selected structures.

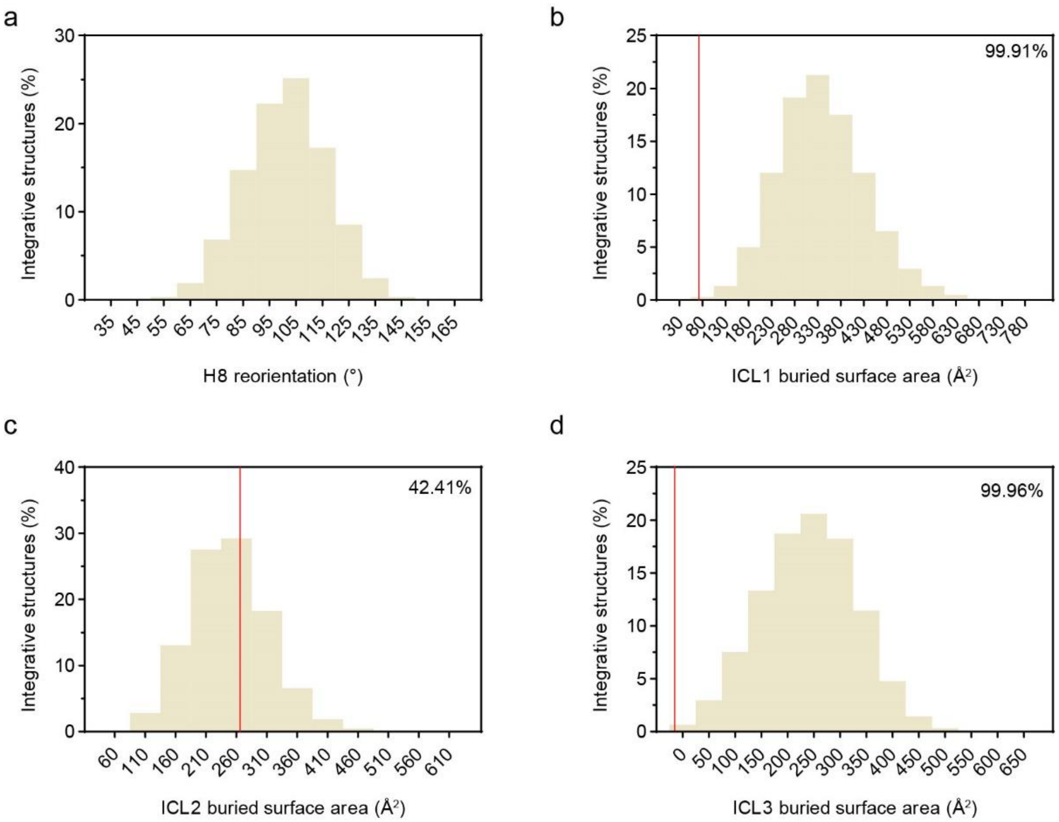

Supplementary Fig. 8 Dynamics of the GLP-1R-Gs interface in integrative structures. a Distribution of the angle of H8 reorientation in integrative structures relative to the H8 in the complete cryo-EM structure. **b-d** Distribution of the buried surface areas of ICL1 (**b**), ICL2 (**c**), and ICL3 (**d**) at the receptor-G_s interface in 30,000 integrative structures. The corresponding buried surface area in the complete cryo-EM structure is annotated by a red line.

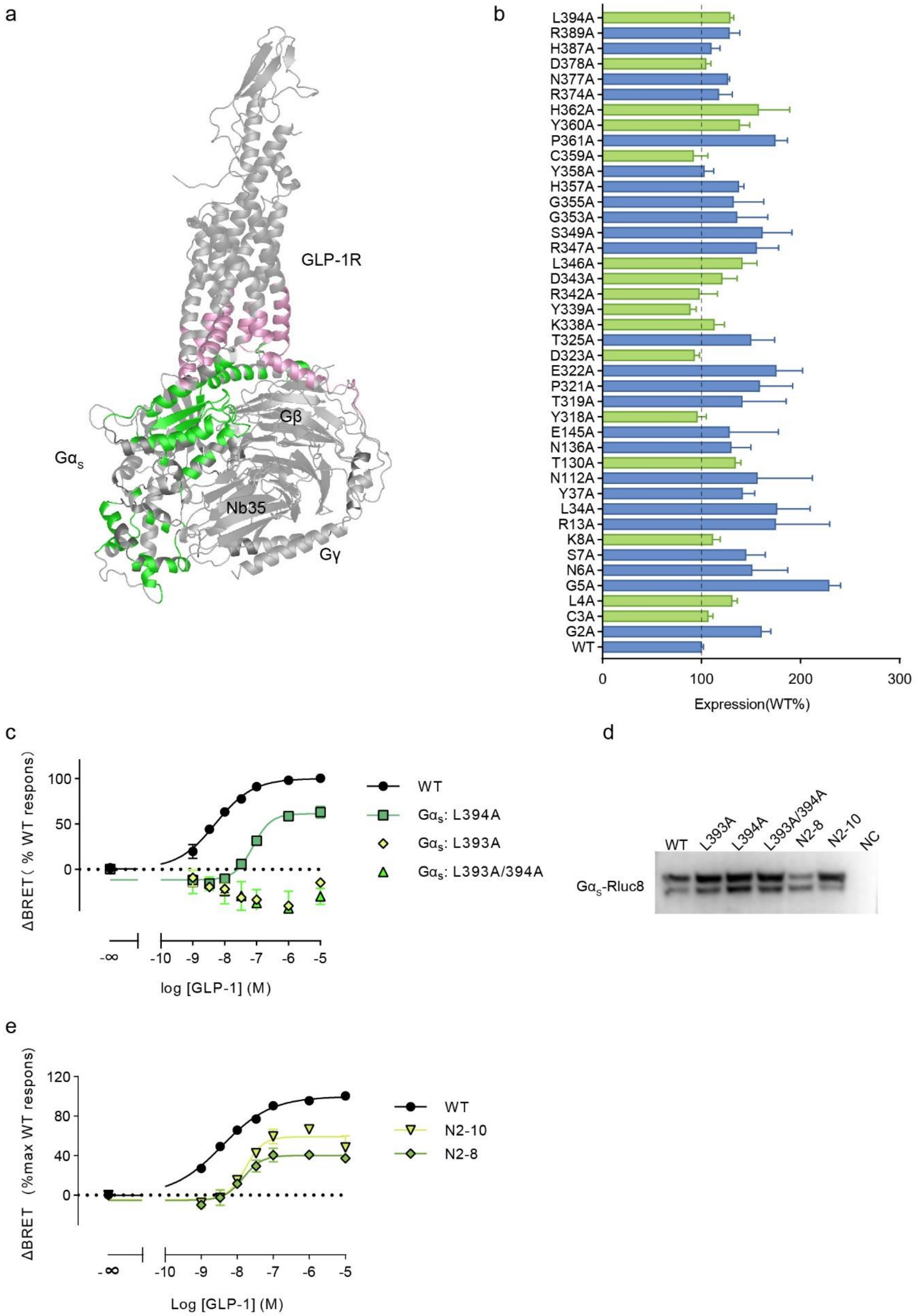
a
GLP-1R
Gβ
Gαs
Nb35
Gγ
b
L394A
R389A
H387A
D378A
N377A
R374A
H362A
Y360A
P361A
C359A
Y358A
H357A
G355A
G353A
S349A
R347A
L346A
D343A
R342A
Y339A
K338A
T325A
D323A
E322A
P321A
T319A
Y318A
E145A
N136A
T130A
N112A
Y37A
L34A
R13A
K8A
S7A
N6A
G5A
L4A
C3A
G2A
WT
0
100
200
300
Expression(WT%)
c
ΔBRET (% WT respons)
100
50
0
-∞
-10
-9
-8
-7
-6
-5
log [GLP-1] (M)
WT
Gαs: L394A
Gαs: L393A
Gαs: L393A/394A
d
WT
L393A
L394A
L393A/394A
N2-8
N2-10
NC
Gαs-Rluc8
e
ΔBRET (%max WT respons)
120
80
40
0
-∞
-10
-9
-8
-7
-6
-5
Log [GLP-1] (M)
WT
N2-10
N2-8

Supplementary Fig. 9 Identification of Gα_s residues important for GLP-1R-mediated G protein activation. a Distribution of the 93 unique GLP-1R residues (pink) and 145 unique Gα_s contact residues (green) making contacts at the interface only present in the integrative structures. The contact residues are labeled in the complete cryo-EM structure. **b** Expression of Gα_s mutants is measured by the luminescence of Rluc8 fused into Gα_s. **c** Concentration-response curves determined by the BRET2-based G protein activation assay for single and double mutants of Gα_s. **d** Western blot of Gα_s-Rluc8 mutants showing normal expression after mutation or truncation. **e** Concentration-response curves determined by the BRET2-based G protein activation assay for the Gα_s truncation mutants of the first 2-8 and 2-10 residues.

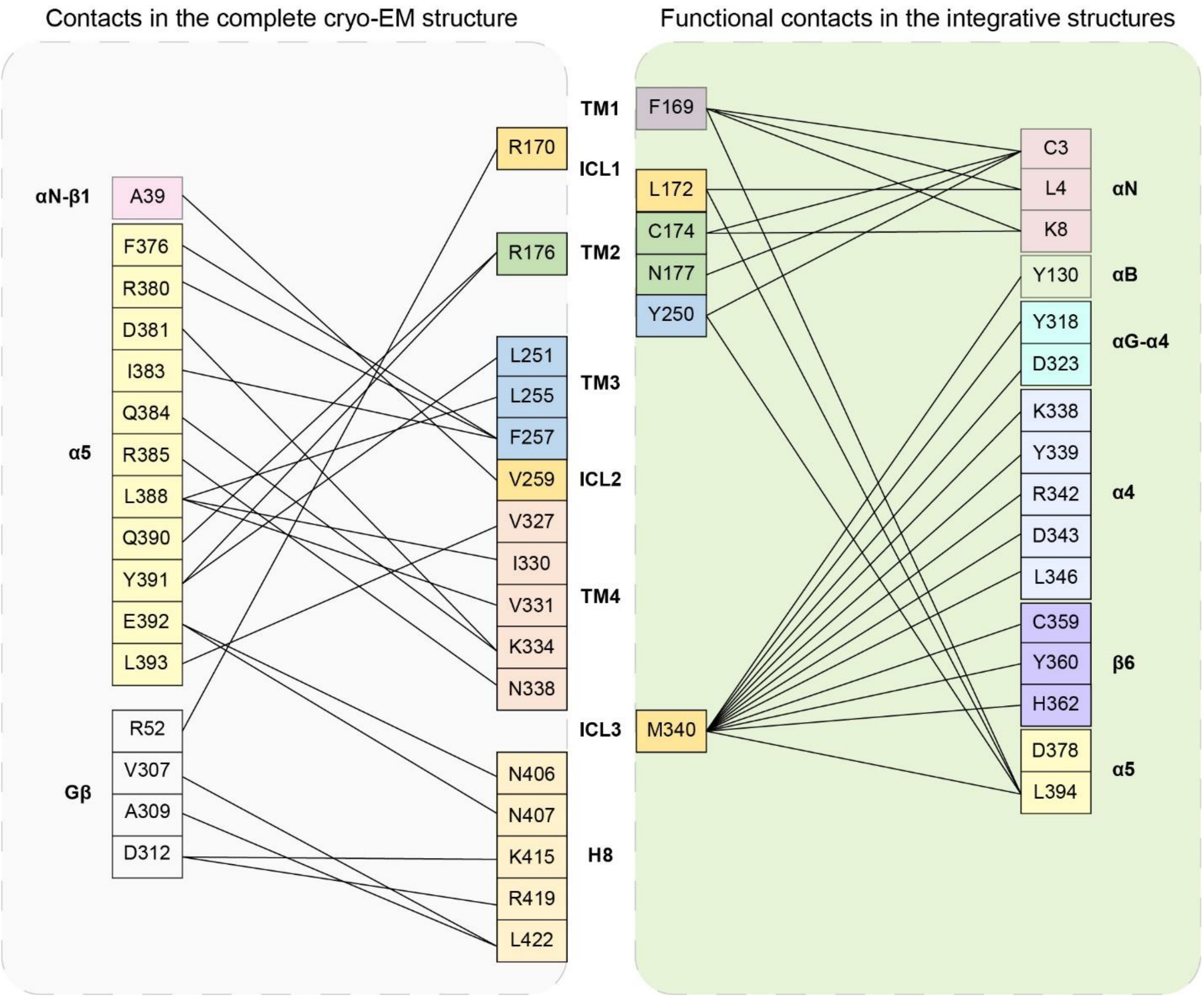

Supplementary Fig. 10 Contacts present in the complete cryo-EM structure (left) and experimentally verified functional contacts present in the integrative structures (right). Only side-chain contacts are shown here. 21 contacts are observed in the complete cryo-EM structure (left) and 24 functional contacts in the integrative structures (right). Residues in different domains are in different color codes.

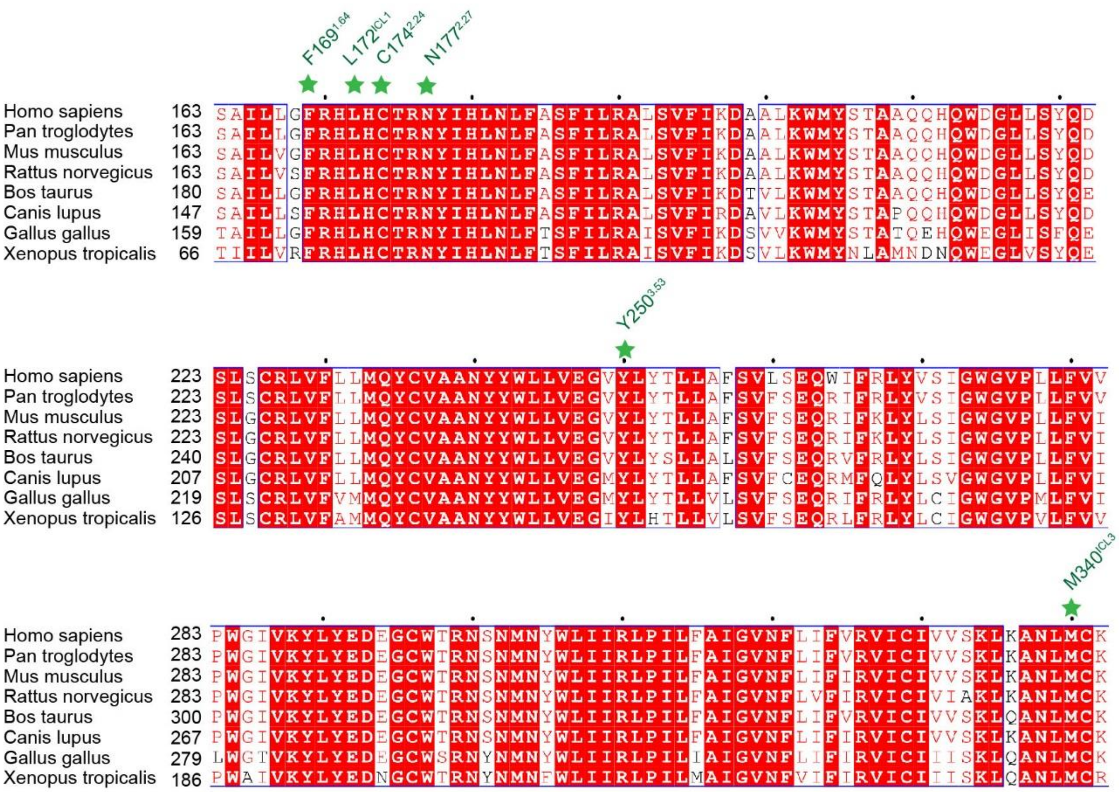

Supplementary Fig. 11 Conservativeness of the six functional GLP-1R residues across species. Multiple sequence alignment of GLP-1R across different species. The six functional residues identified in this work are labeled as green stars.

a

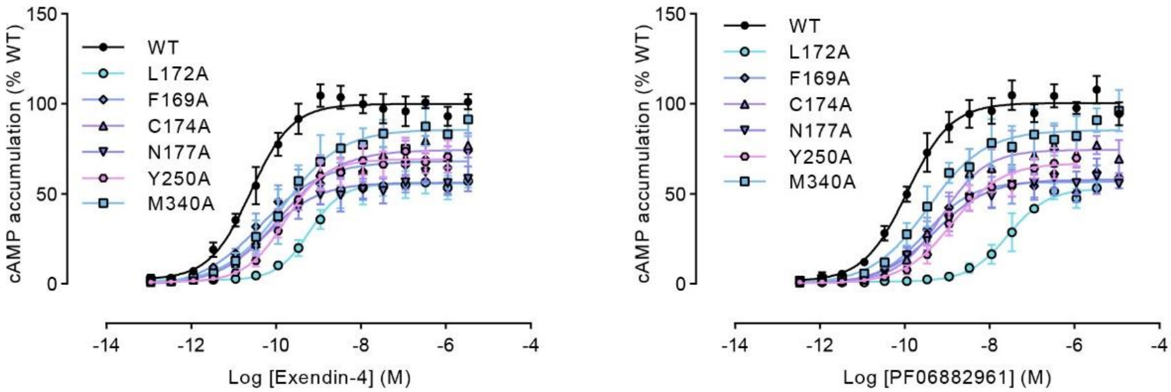

b

	Exendin-4 pEC_{50}	PF06882961 pEC_{50}
WT	10.6±0.08	9.94±0.08
L172A	9.22±0.08	7.57±0.08
F169A	10.35±0.26	9.50±0.15
C174A	9.92±0.11	9.13±0.12
N177A	10.2±0.13	9.29±0.13
Y250A	9.79±0.08	8.88±0.10
M340A	9.85±0.17	9.45±0.15

Supplementary Fig. 12 GLP-1R-mediated cAMP signaling induced by different agonists. Concentration-response curves (a) and EC_{50} measurement (b) for mutant and WT receptor-mediated cAMP signaling by exendin-4 (left) or PF06882961 (right).

Supplementary Method

Integrative structure determination of the GLP-1 receptor-G_s complex

Integrative structure determination of the human GLP-1 receptor-G_s complex proceeded through four stages (Supplementary Figure 4; Supplementary Table 3)[1,2]: (1) Gathering data from multiple sources, (2) Representing the model system and translating data into spatial restraints, (3) Sampling the configurations with good scores and (4) Analyzing and validating the ensemble. The integrative structure modeling protocol (stages 2-4) was scripted using the Python Modelling Interface (PMI) package based on the open-source Integrative Modeling Platform (IMP) package[1] version 2.12.0 (*https://integrativemodeling.org*). Input data, modeling scripts, and output results are available at *https://github.com/salilab/GLP1R-Gs*. The representative integrative structures have been deposited at PDB-Dev (*https://pdb-dev.wwpdb.org/*)[3], with the PDB ID code PDBDEV_00000200.

1.1 Stage 1: Gathering data from multiple sources

We gathered information on the model system, including experimental data, statistical inferences, physical principles, and prior models. We identified 459 highly reproducible intra- and inter-molecular BS3, DSG, EDC, and KArGO cross-links using mass spectrometry (Supplementary Table 2). These cross-links allowed us to determine the spatial proximities and conformations of the six subunits. Prior models of individual subunits relied on their atomic structures determined by cryo-EM (PDB code 6X18[4]). Spatial positions of the GLP-1R transmembrane domain informed about its proximities to the membrane. Steric effects and sequence connectivities were also considered for all subunits.

1.2 Stage 2: Representing the model system and translating data into spatial restraints

Information about the modeled system (above) can in general be used for defining the representation of the model system and the scoring function that guides sampling of alternative structural models, limiting sampling, filtering of representative structures obtained by sampling, and final validation of the structures. The representation of the GLP-1R-G_s complex relied primarily on the complete cryo-EM structure, which is the centroid of 100 structural models built by filling in density missing regions in the cryo-EM structure (Methods). The model was represented using seven rigid bodies consisting of multiple beads. The helical bundles and β-sheets from six subunits with satisfied cross-links were constrained as individual rigid bodies

based on the complete cryo-EM structure. Short structural segments of up to 16 residues linking rigid bodies and unstructured loops of up to 40 residues were represented as flexible strings of beads. Each bead represented a single residue, with the coordinates of the corresponding Cα atoms. In a rigid body, the relative distances between beads were constrained during conformational sampling. In a flexible string, the beads were restrained by the chemical cross-links, protein-membrane interactions, excluded volume, and sequence connectivity.

(1) Cross-link restraints: The 392 intra- and 67 intermolecular links were used to restrain the distances between the cross-linked residues using a Bayesian scoring function[5,6]. Each cross-linker had an upper bound on the distance, calculated as the Euclidean distance plus a tolerance of 10 Å to account for the lengths of cross-linked sidechains and the uncertainty of modeling the backbone positions[7]. Thus, the maximum distances for the BS3, DSG, EDC, and KArGO cross-links were 35 Å, 30 Å, 25 Å, and 40 Å, respectively. The cross-link restraint only considered the least violated distance for the cross-linked residues. For each linker, intra- and intermolecular links were combined into a single restraint due to the limited amount of cross-linking data.

(2) Membrane restraints: The spatial positions of the GLP-1R transmembrane domain and the hydrophobic thickness of the membrane (29.4 Å) were calculated using the PPM webserver (*https://opm.phar.umich.edu/ppm_server*)[8]. The center of the membrane was determined as the average z coordinates of Cα atoms of the Glp-1R transmembrane domains. The membrane restraints were applied to domains above, inside, and below the membrane using a harmonic score to localize beads within the transmembrane domains and to prevent beads in the non-transmembrane domains from penetrating the membrane.

(3) Excluded volume restraints: The excluded volume restraints were applied to each bead, using the statistical relationship between the volume and the number of residues that it covers [9–11].

(4) Sequence connectivity restraints: The sequence connectivity restraints were applied using a harmonic upper bound on the distance between consecutive beads in each subunit, with a threshold distance equal to twice the sum of the radii of the two connected beads. The bead radius was calculated from the excluded volume of the corresponding bead, assuming standard protein density[5, 10, 12, 13].

1.3 Stage 3: Sampling the configurations with good scores

We used configurational sampling to produce the ensemble of models that satisfies the restraints. We searched for the positions of the rigid bodies and beads using replica exchange Gibbs sampling based on the Metropolis Monte Carlo algorithm[14]. The Monte Carlo moves included random translations and rotations of rigid bodies (up to 1 Å and 0.01 radians, respectively) and random translations of individual beads in the flexible segments (up to 2 Å). We performed 100 independent sampling calculations with a temperature range of 1.0–5.0, each starting with a random initial configuration. The model coordinates were saved every 10 Gibbs sampling steps, each consisting of a cycle of Monte Carlo steps that moved every rigid body and flexible bead once. After about 7 days of configurational sampling on 72 computational cores, we produced a total of 15,000,000 models. The sampling yielded 844,020 good-scoring structures that sufficiently satisfied the input restraints.

1.4 Stage 4: Analyzing and validating the ensemble

To estimate the precision and accuracy of the output structures, we need to analyze the input information and the structures themselves. This helps us to detect any inconsistent or missing information and suggest more informative future experiments.

The analysis and validation protocol we used has been demonstrated in previous publications[15,16]. This assessment process began by testing the thoroughness of the structural sampling, followed by structural clustering of the models and estimating their precision based on the variability in the ensemble of good-scoring models. We then quantified the fit of the structure to the input information and assessed the structure using data that was not used to compute it. These validations are based on the nascent wwPDB effort to archive, validate, and disseminate integrative structure models[2].

(1) Thoroughness of the configurational sampling

To test the thoroughness of the configurational sampling, we performed four convergence tests as follows.

First, three clusters were obtained by threshold-based clustering using an RMSD threshold of 21 Å. Cluster 0 containing 89% (>80%) of the models was considered the dominant cluster. The sampling precision is 21 Å, defined as the average bead RMSD between the models within the cluster and its corresponding centroid in the finest clustering for which each sample contributes models proportionally to its size (considering both significance and magnitude of the difference)

and for which a sufficient proportion of all models occur in sufficiently large clusters. This test involved clustering models from both samples and comparing the proportions of models from each sample in each cluster. The χ2-test p-value is 1.00 (>0.05) indicating no statistically significant difference between the sample distributions among all clusters. The Cramer's V is 0.00 (<0.10) indicating an insignificant magnitude of difference between the sample distributions[17] (Supplementary Figure 5a). A comparison of two integrative structures considered all beads representing subunits of the GLP-1R-G_s complex.

Second, we randomly selected 30,000 structures of the GLP-1R-G_s complex out of the total 753,840 good-scoring models in cluster 0, resulting in 12,225 and 17,774 structures in sample A and B respectively (Supplementary Figure 5b-c). The scores do not continue to improve as more structures are computed, essentially independently of each other (Supplementary Figure 5d).

Third, the selected structures in sample A and B satisfied the data equally well (Supplementary Figure 5e). The non-parametric Kolmogorov–Smirnov two-sample test (two-sided)[17] indicates that the difference between the two score distributions is insignificant, the magnitude of the difference is small, as demonstrated by the Kolmogorov–Smirnov two-sample test statistic, D, of 0.11 (Supplementary Figure 5e).

Fourth, we compared two localization probability density maps obtained for structures in samples A and B (Supplementary Figure 5f). A localization probability density map defines the probability of any voxel being occupied by a specific protein in a set of model densities, which in turn are obtained by convolving superposed models with a Gaussian kernel. The average cross-correlation coefficient between the two maps is 0.997, indicating that the position of GLP-1R-G_s in the two samples is nearly identical. Therefore, all four sampling tests indicate that the sampling was exhaustive at 16.9 Å precision. The caveat is that passing these tests is only necessary but not sufficient as evidence of thorough sampling; a positive outcome of the tests may be misleading if, for example, the landscape contains only a narrow, and thus difficult to find, a pathway to the pronounced minimum corresponding to the correct structure.

(2) Clustering and structure precision

The precision of a component position can be quantified by its variation in an ensemble of superposed good-scoring structures. It can also be visualized by the localization probability density for each of the structural components. As described above, integrative structure determination of the GLP-1R-G_s resulted in effectively a single solution, at the precision of 16.9

Å. The centroid structure that minimizes the sum of the RMSD values against all other structures in the ensemble

(3) Fitting to data used to construct the model

Here, we consider a cluster satisfies a cross-link if the cross-linked distance in any individual model in the cluster is less than the maximum threshold (i.e., the maximum length of the assessed cross-linkers). The satisfaction rates for different types of cross-linkers are all significantly higher for the integrative structures (85.7%-99.4%) than for the complete cryo-EM structure (75.9%-90.8%) (Supplementary Table 4). The remainder of the restraints is harmonic, with a specified standard deviation. A restraint is satisfied by a cluster of structures if the restrained distance in any structure in the cluster (considering restraint ambiguity) is violated by less than 3 standard deviations, specified for the restraint. Most of the violations are small and can be rationalized by local structural fluctuations, coarse-grained representations of some GLP-1R-G_s domains and/or finite sampling.

(4) Assessing the model by data not used to construct the model

All 30,000 coarse-grained integrative structures were back-mapped to atomic ones using MODELLER 10.2[18]. We generated atomic structures by completing side chains for all residue beads (*ie*., Cα atoms), followed by Molecular dynamics refinements. For each coarse-grained structure, we determined its optimal atomic structure as the one with the lowest Discrete Optimized Protein Energy (DOPE) score. From the 30,000 atomic integrative structures, we screened key residue contacts between the GLP-1 receptor and G proteins that were only observed in the integrative structures, yet absent in the cryo-EM structure. We then validated them by alanine-scanning mutagenesis coupled with pharmacological assays (Methods).

References

1. Russel, D. *et al.* Putting the pieces together: integrative modeling platform software for structure determination of macromolecular assemblies. *PLoS Biol.* **10**, e1001244 (2012).
2. Sali, A. *et al.* Outcome of the First wwPDB Hybrid/Integrative Methods Task Force Workshop. *Structure* **23**, 1156–1167 (2015).
3. Vallat, B., Webb, B., Westbrook, J., Sali, A. & Berman, H. M. Archiving and disseminating integrative structure models. *J. Biomol. NMR* **73**, 385–398 (2019).
4. Zhang, X. *et al.* Differential GLP-1R Binding and Activation by Peptide and Non-peptide Agonists. *Mol. Cell* **80**, 485–500.e7 (2020).

5. Shi, Y. *et al.* Structural characterization by cross-linking reveals the detailed architecture of a coatomer-related heptameric module from the nuclear pore complex. *Mol. Cell. Proteomics* **13**, 2927–2943 (2014).
6. Rieping, W., Habeck, M. & Nilges, M. Inferential structure determination. *Science* **309**, 303–306 (2005).
7. Surface Accessibility and Dynamics of Macromolecular Assemblies Probed by Covalent Labeling Mass Spectrometry and Integrative Modeling. *Analytical chemistry*. **89(3),**1459-1468 (2017).
8. Lomize, M. A., Pogozheva, I. D., Joo, H., Mosberg, H. I. & Lomize, A. L. OPM database and PPM web server: resources for positioning of proteins in membranes. *Nucleic Acids Res.* **40**, D370–6 (2012).
9. Alber, F. *et al.* The molecular architecture of the nuclear pore complex. *Nature* **450**, 695–701 (2007).
10. LoPiccolo, J. *et al.* Assembly and Molecular Architecture of the Phosphoinositide 3-Kinase p85α Homodimer. *J. Biol. Chem.* **290**, 30390–30405 (2015).
11. Shen, M.-Y. & Sali, A. Statistical potential for assessment and prediction of protein structures. *Protein Sci.* **15**, 2507–2524 (2006).
12. Kim, S. J. *et al.* Integrative structure and functional anatomy of a nuclear pore complex. *Nature* **555**, 475–482 (2018).
13. Algret, R. *et al.* Molecular architecture and function of the SEA complex, a modulator of the TORC1 pathway. *Mol. Cell. Proteomics* **13**, 2855–2870 (2014).
14. Fernandez-Martinez, J. *et al.* Structure and Function of the Nuclear Pore Complex Cytoplasmic mRNA Export Platform. *Cell* **167**, 1215–1228.e25 (2016).
15. Saltzberg, D. J. *et al.* Using *Integrative Modeling Platform* to compute, validate, and archive a model of a protein complex structure. *Protein Science* **30**, 250–261 (2021).
16. Viswanath, S., Chemmama, I. E., Cimermancic, P. & Sali, A. Assessing Exhaustiveness of Stochastic Sampling for Integrative Modeling of Macromolecular Structures. *Biophysical Journal* **113** 2344–2353 (2017).
17. Siegel, S. & John Castellan, N. *Nonparametric Statistics for the Behavioral Sciences*. (McGraw-Hill Humanities, Social Sciences & World Languages, 1988).
18. Webb, B. & Sali, A. Comparative Protein Structure Modeling Using MODELLER. *Curr. Protoc. Bioinformatics* **54**, (2016).
